# Safety and feasibility of single-incision laparoscopic distal gastrectomy in overweight and obese gastric cancer patients: a propensity score-matched analysis

**DOI:** 10.1007/s10120-024-01530-5

**Published:** 2024-07-18

**Authors:** Eunju Lee, Yun-Suhk Suh, Mira Yoo, Duyeong Hwang, So Hyun Kang, Sangjun Lee, Young Suk Park, Sang-Hoon Ahn, Seong-Ho Kong, Do Joong Park, Hyuk-Joon Lee, Hyung-Ho Kim, Han-Kwang Yang

**Affiliations:** 1 Department of Surgery, Seoul National University Bundang Hospital, 82, Gumi-Ro 173Beon-Gil, Bundang-Gu, Seongnam-Si, Gyeonggi-Do Republic of Korea; 2 Department of Surgery, Chung-Ang University Gwangmyeong Hospital, Gwangmyeong-Si, Republic of Korea; 3 Department of Surgery, Seoul National University College of Medicine, Seoul, Republic of Korea; 4 Department of Surgery, Kyung Hee University Hospital at Gangdong, Seoul, Republic of Korea; 5 Department of Surgery, Seoul National University Hospital, Seoul, Republic of Korea; 6 Department of Surgery, Chung-Ang University College of Medicine, Seoul, Republic of Korea

**Keywords:** Stomach Neoplasms, Gastrectomy, Minimally Invasive Surgical Procedures, Obesity

## Abstract

**Background:**

The technical challenges and safety concerns of single-incision laparoscopic gastrectomy for overweight and obese gastric cancer patients remain unclear. This study aimed to evaluate the safety and feasibility of single-incision laparoscopic distal gastrectomy (SIDG) compared to multiport laparoscopic distal gastrectomy (MLDG) in overweight and obese gastric cancer patients.

**Methods:**

This study retrospectively analyzed overweight and obese patients (body mass index ≥ 25 kg/m^2^) and pathologic stage T1 primary gastric adenocarcinoma treated with either SIDG or MLDG. The SIDG and MLDG groups were propensity score matched at a 1:2 ratio using age, sex, height, body weight, American Society of Anesthesiologists classification, year of surgery, pathologic N stage, and anastomosis method as covariates.

**Results:**

After 1:2 matching, the study included patients who underwent SIDG (n = 179) and MLDG (n = 358). No significant difference in the number of retrieved lymph nodes was found between the SIDG and MLDG groups (52.8 ± 19.3 vs. 53.9 ± 21.0, *P* = 0.56). Operation times were significantly shorter in the SIDG group (170.8 ± 60.0 min vs. 186.1 ± 52.6 min, *P* = 0.004). The postoperative hospital length of stay was comparable between the 2 groups (SIDG: 5.9 ± 3.4 days vs. MLDG: 6.3 ± 5.1 days, *P* = 0.23), as was postoperative complication rate (SIDG: 13.4% vs. MLDG: 12.8%, *P* = 0.89).

**Conclusions:**

SIDG was shown to be as safe and feasible as MLDG for overweight and obese gastric cancer patients, with comparable early postoperative complication rates without compromising operation time compared to MLDG.

**Supplementary Information:**

The online version contains supplementary material available at 10.1007/s10120-024-01530-5.

## Background

Laparoscopic gastrectomy has been established as a safe and effective approach for gastric cancer and is widely accepted worldwide [1–4]. The introduction of transumbilical single-incision laparoscopic distal gastrectomy (SIDG) in 2011 marked a significant advancement in surgical techniques, combining the advantages of minimally invasive surgery with the potential for improved patient outcomes [5]. Several studies have provided substantial evidence supporting the safety and efficacy of SIDG [5, 6]. Previous research has highlighted several benefits of this procedure, including reduced postoperative pain, shorter operation time, faster recovery after surgery, and reduced manpower requirements [7–11]. The reported advantages of reduced port surgery, along with the standardization of surgical techniques and advancements in surgical instruments, are garnering increasing interest in the field [12, 13]. However, there are still concerns about the technical difficulty of the procedure, particularly in obese patients [12].

In Asian countries, including Korea, obesity rates have been rising rapidly. In 2019, the adult obesity rate in Korea was reported to be 36.3%, with a higher prevalence in men (46.2%) than in women (27.3%) [14]. The increasing prevalence of obesity presents challenges for single-incision gastrectomy due to the difficulty in maintaining an adequate field of view, primarily due to excess fat tissue hindering visualization during the procedure [15]. While there is limited literature available on the application of reduced-port gastrectomy in obese gastric cancer patients [16], we found no studies on the use of single-incision laparoscopic gastrectomy in obese patients. Therefore, this study aimed to investigate the safety and feasibility of SIDG in overweight and obese patients diagnosed with gastric cancer.

## Methods

We conducted a retrospective review of patients who underwent totally laparoscopic distal gastrectomy with curative intent for primary gastric adenocarcinoma between July 2011 and March 2021 at Seoul National University Bundang Hospital and between March 2014 and November 2017 at Seoul National University Hospital. The study included patients who underwent either SIDG or conventional multiport laparoscopic distal gastrectomy (MLDG). The study population consisted of patients with a body mass index (BMI) ≥ 25 kg/m^2^ and early gastric cancer (EGC) defined by a pathologic T1 stage.

Patients with distant metastasis and those who underwent reduced port surgery with 2–4 ports were excluded. Patients who received neoadjuvant chemotherapy or underwent the resection of other organs during the operation, except for prophylactic cholecystectomy and appendectomy, were also excluded.

The criteria for defining overweight and obesity in Asians are different than the global standards established by the World Health Organization [17]. According to the global criteria, the cut-off values distinguishing normal weight from overweight and overweight from obesity are 25 kg/m^2^ and 30 kg/m^2^, respectively. However, in the Asian population, previous reports suggested an increased risk of cardiovascular events in patients with a BMI of ≥ 27.5 kg/m^2^, leading to the consideration of lower cut-off values of 23 kg/m^2^ to distinguish between normal weight and overweight, and 27.5 kg/m^2^ to differentiate between overweight and obesity [17, 18]. Thus, we categorized patients into 3 distinct groups: BMI group A had a BMI of 25 kg/m^2^ or above but less than 27.5 kg/m^2^, BMI group B had a BMI of 27.5 kg/m^2^ or above but less than 30 kg/m^2^, and BMI group C had a BMI of 30 kg/m^2^ or above.

The SIDG and MLDG study groups were propensity score matched at a 1:2 ratio. The propensity score of each patient was estimated by logistic regression using statistical analysis in the MatchIt package in R version 4.1.0. [19]. The matching variables included age, sex, height, body weight, American Society of Anesthesiologists classification, year of surgery, pathologic N stage, and the method of anastomosis. Nearest neighbor matching method of caliper 0.25 was used for propensity score-matching (PSM). The primary endpoint of this study was early postoperative complications within 30 days. The severity of complications was assessed using the Clavien-Dindo classification and Comprehensive Complication Index (CCI) [20, 21]. The secondary endpoints included operative outcomes, such as operation time, intraoperative transfusion rate, the number of retrieved lymph nodes, and the postoperative hospital length of stay.

**Fig. 1:**
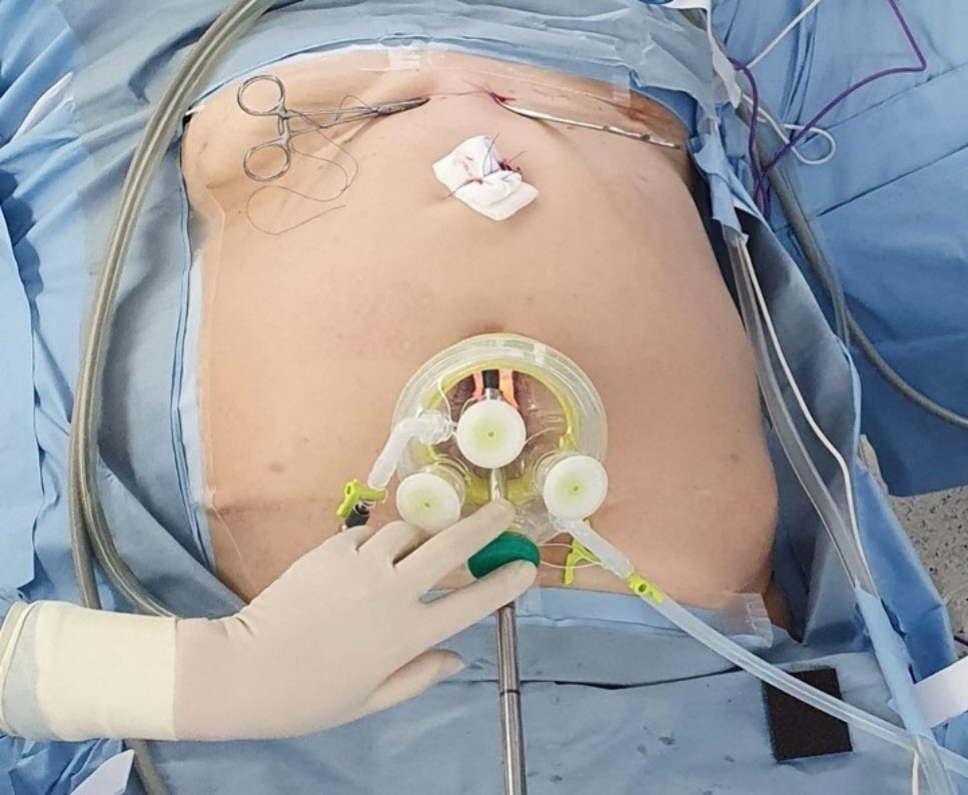
Surgical field photograph taken during single-incision distal gastrectomy

During SIDG, the patients were positioned in the lithotomy position, and the surgeon sat between the patients’ legs to perform the surgery through a transumbilical port of approximately 3–4 cm in size, which could be extended for safe specimen retrieval (Fig. 1). During the procedure, the surgeon determined whether to use of a laparoscopic articulating device (ArtiSential Fenestrated Forceps, ArtiSential Dissector, ArtiSential Needle Holder; Livsmed, Seongnam, Korea) or a self-intracorporeal retractor (FJ Clip; Charmant, Sabae, Japan) for achieving optimal visualization and traction. Detailed procedures for SIDG have been outlined in prior publications [22–24]. During MLDG, the patients were positioned in the supine position. Throughout the procedure, the surgeon consistently stood on the patient’s right side. Basic surgical procedures for MLDG are described in prior studies [2, 25].

Distal gastrectomy with D1 + lymph node dissection (LND) was mainly performed for both SIDG and MLDG according to the gastric cancer treatment guidelines [25, 26]. The standard procedure for SIDG and reconstruction, such as Billroth I and Billroth II, was conducted similarly to previously described methods [22, 23]. Patients in both the SIDG and MLDG groups received the same perioperative care.

A cost analysis was conducted to evaluate whether there were cost differences between SIDG and MLDG. Data on total hospital costs, operation and procedure costs, and treatment material costs associated with hospitalization for surgery were obtained from the Insurance and Assessment Department of each institution. The Mann–Whitney U test was used to examine differences in the distribution of each cost, and results were presented as the median with interquartile range (IQR) between the 25th and 75th percentiles. The indirect cost attributed to the assistant surgeon’s workload data was obtained from the Korean Health Insurance Review and Assessment Service. The relative value score calculations were employed to determine the indirect cost for the assistant surgeon during distal gastrectomy with LND. Costs were converted from Korean Won to USD at an exchange rate of 1385 KRW–1 USD, based on the rate on May 31, 2024.

Statistical analyses were performed using R version 4.1.0 and Python version 3.11. Categorical variables were analyzed using the chi-square test or Fisher’s exact test, while continuous variables were analyzed using either the t-test or the Mann–Whitney U test. Continuous variables are presented as the mean ± standard deviation. If the Mann–Whitney U test was used, and the results were presented as the median with IQR. A p-value of less than 0.05 was considered statistically significant. The study was approved by the Institutional Review Board at Seoul National University Bundang Hospital (IRB number: B-2203-744-105).

## Results

**Fig. 2:**
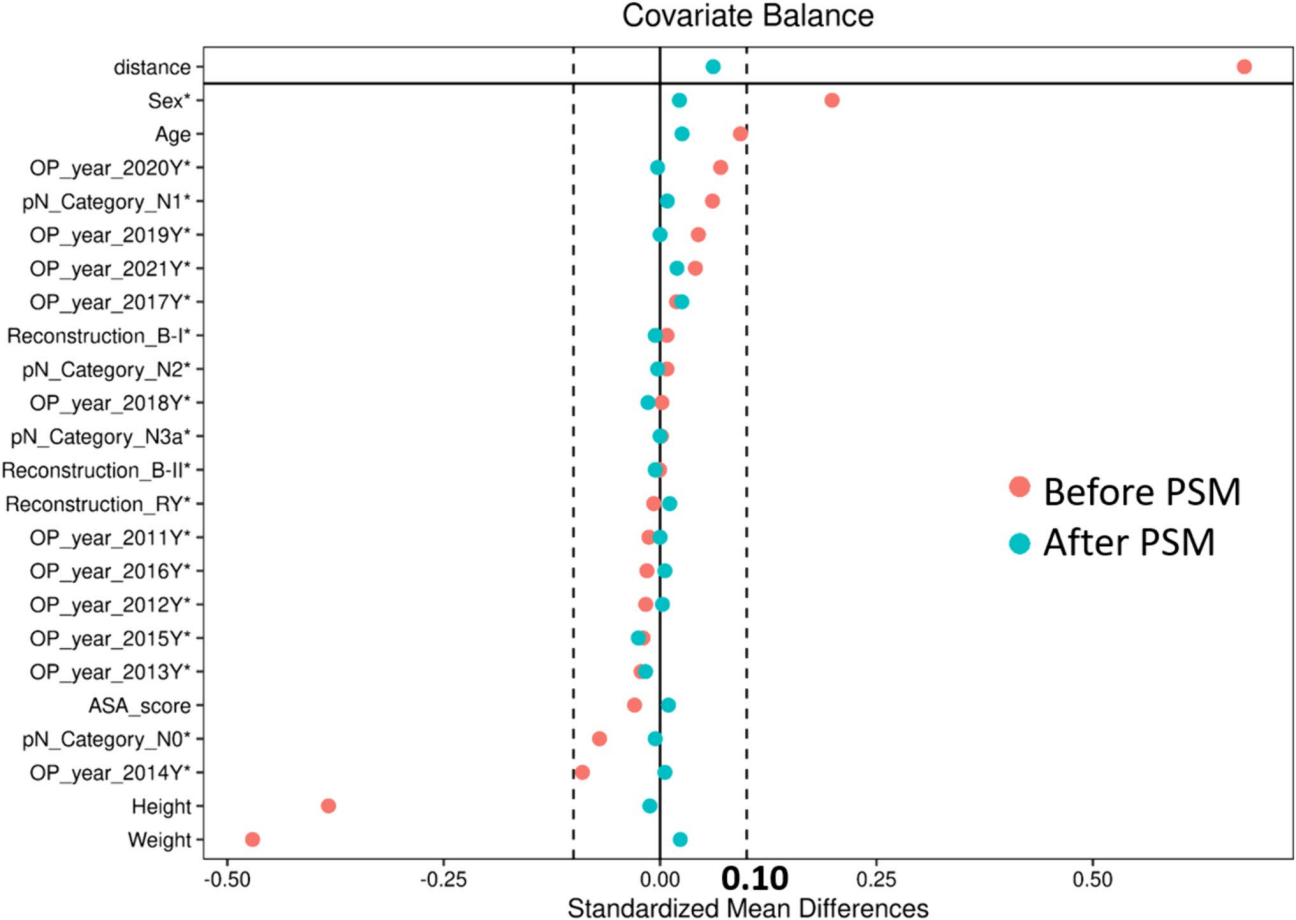
The covariate balance before and after 1:2 propensity score matching between single-incision distal gastrectomy (SIDG) and multiport laparoscopic distal gastrectomy (MLDG) groups

A total of 1109 patients were included in this study, with 179 patients in the SIDG group and 930 patients in the MLDG group. After 1:2 PSM, the SIDG group consisted of 179 patients, and the MLDG group consisted of 358 patients. The standardized mean difference for all matching variables was less than 0.1 after PSM, indicating successful matching (Fig. 2).

Table 1 shows the clinicopathologic characteristics of the SIDG and MLDG groups before and after 1:2 PSM. Before PSM, the MLDG group had a higher proportion of male patients, greater height and weight, and higher BMIs than the SIDG group. However, after 1:2 PSM, there was no difference in age, sex, height, weight, BMI, or ASA classification between the two groups. Before matching, the year of surgery was statistically significantly different between the two groups (*P* < 0.001). In the case of SIDG, which was introduced relatively recently, the number of cases inevitably increased over time. Therefore, the year of surgery was included as a matching variable to mitigate bias from changes in the surgical technique and perioperative management. After PSM, the year of surgery was well balanced between the two groups. (*P* = 0.96).

**Table 1:** Comparison of clinicopathologic characteristics in single-incision distal gastrectomy (SIDG) and multiport laparoscopic distal gastrectomy (MLDG) before and after 1:2 propensity score matching

	Before matching			After matching		
	SIDG (N = 179)	MLDG (N = 930)	*P* value	SIDG (N = 179)	MLDG (N = 358)	*P* value
Sex (M:F)	93:86 (male 52.0%)	668:262 (male 71.8%)	< 0.001	93:86 (male 52.0%)	194:164 (male 54.2%)	0.69
Age (years)	61.8 ± 11.4	60.8 ± 11.2	0.25	61.8 ± 11.4	61.6 ± 11.4	0.78
Height (cm)	160.8 ± 8.8	164.2 ± 9.0	< 0.001	160.8 ± 8.8	161.0 ± 9.3	0.90
Weight (kg)	70.5 ± 8.7	74.6 ± 9.7	< 0.001	70.5 ± 8.7	70.3 ± 9.2	0.81
BMI^a^ (kg/m^2^)	27.2 ± 1.8	27.6 ± 2.3	0.006	27.2 ± 1.8	27.0 ± 1.9	0.47
The number of patients according to BMI subgroup						
25 ≤ BMI < 27.5	112 (62.6%)	535 (57.5%)	0.01	112 (62.6%)	235 (65.6%)	0.23
27.5 ≤ BMI < 30	58 (32.4%)	275 (29.6%)		58 (32.4%)	95 (26.5%)	
BMI ≥ 30	9 (5.0%)	120 (12.9%)		9 (5.0%)	28 (7.8%)	
ASA^b^ classification			0.68			0.56
I	54 (30.2%)	256 (27.5%)		54 (30.2%)	106 (29.6%)	
II	111 (62.0%)	612 (65.8%)		111 (62.0%)	230 (64.2%)	
III	14 (7.8%)	60 (6.5%)		14 (7.8%)	20 (5.6%)	
IV	0 (0.0%)	2 (0.2%)		0 (0.0%)	2 (0.6%)	
Year of operation			< 0.001			0.96
2011	0 (0.0%)	12 (1.3%)		0 (0.0%)	0 (0.0%)	
2012	3 (1.7%)	31 (3.3%)		3 (1.7%)	5 (1.4%)	
2013	8 (4.5%)	62 (6.7%)		8 (4.5%)	22 (6.1%)	
2014	8 (4.5%)	125 (13.4%)		8 (4.5%)	14 (3.9%)	
2015	24 (13.4%)	143 (15.4%)		24 (13.4%)	57 (15.9%)	
2016	30 (16.8%)	170 (18.3%)		30 (16.8%)	58 (16.2%)	
2017	29 (16.2%)	133 (14.3%)		29 (16.2%)	49 (13.7%)	
2018	20 (11.2%)	102 (11.0%)		20 (11.2%)	45 (12.6%)	
2019	21 (11.7%)	68 (7.3%)		21 (11.7%)	42 (11.7%)	
2020	26 (14.5%)	70 (7.5%)		26 (14.5%)	53 (14.8%)	
2021	10 (5.6%)	14 (1.5%)		10 (5.6%)	13 (3.6%)	
Tumor size (cm)	2.7 ± 1.5	2.6 ± 1.5	0.36	2.7 ± 1.5	2.7 ± 1.7	0.85
Proximal resection margin (cm)	4.7 ± 2.7	4.7 ± 2.9	0.92	4.7 ± 2.7	4.5 ± 2.7	0.50
Distal resection margin (cm)	5.2 ± 3.2	5.5 ± 3.0	0.18	5.2 ± 3.2	5.2 ± 2.8	0.86
Number of retrieved lymph nodes	52.8 ± 19.3	54.6 ± 21.9	0.26	52.8 ± 19.3	53.9 ± 21.0	0.56
Number of positive lymph nodes	0.4 ± 1.2	0.3 ± 1.1	0.19	0.4 ± 1.2	0.4 ± 1.2	0.98
Lymphatic invasion	31 (17.3%)	118 (12.7%)	0.12	31 (17.3%)	60 (16.8%)	0.97
Venous invasion	1 (0.6%)	12 (1.3%)	0.65	1 (0.6%)	6 (1.7%)	0.50
Perineural invasion	9 (5.0%)	33 (3.6%)	0.47	9 (5.0%)	12 (3.4%)	0.48
Pathologic T stage			0.18			0.69
pT1a	94 (52.5%)	542 (58.3%)		94 (52.5%)	196 (54.7%)	
pT1b	85 (47.5%)	388 (41.7%)		85 (47.5%)	162 (45.3%)	
Pathologic N stage			0.03			0.99
pN0	148 (82.7%)	834 (89.7%)		148 (82.7%)	298 (83.2%)	
pN1	22 (12.3%)	58 (6.2%)		22 (12.3%)	41 (11.5%)	
pN2	7 (3.9%)	29 (3.1%)		7 (3.9%)	15 (4.2%)	
pN3a	2 (1.1%)	9 (1.0%)		2 (1.1%)	4 (1.1%)	
Pathologic TNM stage			0.003			0.45
Stage I	151 (84.4%)	853 (91.7%)		151 (84.4%)	312 (87.2%)	
Stage II	28 (15.6%)	77 (8.3%)		28 (15.6%)	46 (12.8%)	

Continuous variables are presented as the mean ± standard deviation

^a^*BMI* body mass index

^b^*ASA* American Society of Anesthesiologists

No significant differences in pathological outcomes were found between the two groups before and after PSM, including tumor size and proximal and distal resection margins. The number of retrieved lymph nodes did not significantly differ between the SIDG and MLDG groups, either before PSM (SIDG: 52.8 ± 19.3 vs. MLDG: 54.6 ± 21.9, *P* = 0.26) or after (SIDG: 52.8 ± 19.3 vs. MLDG: 53.9 ± 21.0, *P* = 0.56). Similarly, the number of positive lymph nodes was not significantly different between the SIDG and MLDG groups before (SIDG: 0.4 ± 1.2 vs. MLDG: 0.3 ± 1.1, *P* = 0.19) or after PSM (SIDG: 0.4 ± 1.2 vs. MLDG: 0.4 ± 1.2, *P* = 0.98). No significant differences in lymphatic invasion, venous invasion, and perineural invasion were found between the SIDG and MLDG groups before or after PSM. Before PSM, the SIDG group had more cases of pathologic stage II (*P* = 0.003) due to a higher proportion of advanced N stages than the MLDG group. However, there were no significant differences in pathologic stages between the groups after PSM (*P* = 0.45).

Table 2 presents the surgical outcomes and postoperative course of the patients in the SIDG and MLDG groups before and after 1:2 PSM. The mean operation time was significantly shorter in the SIDG group compared to the MLDG group both before (170.8 ± 60.0 min vs. 190.6 ± 53.9 min, *P* < 0.001) and after PSM (170.8 ± 60.0 min vs. 186.1 ± 52.6 min, *P* = 0.004). In the subgroup analysis (Table 3, Supplementary Table 2), a significant difference in operation time was observed between SIDG and MLDG groups within the BMI 25–30 kg/m^2^ category, with SIDG demonstrating a shorter operation time (BMI 25–27.5 kg/m^2^ group: SIDG, 168.6 ± 64.8 vs. MLDG, 182.6 ± 53.6, *P* = 0.047; BMI 27.5–30 kg/m^2^ group: SIDG, 171.9 ± 52.5 vs. MLDG, 195.4 ± 52.0, *P* = 0.008). However, no significant difference in operation time was found in the subgroup with a BMI of 30 kg/m^2^ or higher between the SIDG and MLDG groups before (SIDG group: 191.1 ± 39.4, MLDG group: 192.3 ± 53.1, *P* = 0.95) or after PSM (SIDG group: 191.1 ± 39.4, MLDG group: 183.4 ± 43.8, *P* = 0.64). No significant differences in the type of anastomosis were found between the SIDG and MLDG groups. Roux-en-Y anastomosis was the most frequently used method in both the SIDG (54.7%) and MLDG groups (53.6%), followed by Billroth-I and Billroth-II. Before matching, the SIDG group had a shorter postoperative hospital stay compared to the MLDG group (SIDG: 5.9 ± 3.4 days vs. MLDG: 6.6 ± 5.2 days, *P* = 0.03). However, after PSM, a trend toward shorter postoperative hospital stays in the SIDG group compared to the MLDG group was seen, but there was no statistical difference (SIDG: 5.9 ± 3.4 days vs. MLDG: 6.3 ± 5.1 days, *P* = 0.23). This trend was maintained in the BMI subgroup analysis (Supplementary Table 2).

**Table 2:** Surgical outcomes and postoperative course of single-incision distal gastrectomy (SIDG) and multiport laparoscopic distal gastrectomy (MLDG) before and after 1:2 propensity score matching

	Before matching			After matching		
	SIDG (N = 179)	MLDG (N = 930)	*P* value	SIDG (N = 179)	MLDG (N = 358)	*P* value
Operation time (min)	170.8 ± 60.0	190.6 ± 53.9	< 0.001	170.8 ± 60.0	186.1 ± 52.6	0.004
Intraoperative transfusion	0 (0.0%)	2 (0.2%)	> 0.99	0 (0.0%)	1 (0.3%)	> 0.99
Concomitant cholecystectomy and appendectomy	6 (3.4%)	27 (2.9%)	0.93	6 (3.4%)	10 (2.8%)	0.93
Anastomosis			0.97			0.97
Billroth I	43 (24.0%)	216 (23.2%)		43 (24.0%)	88 (24.6%)	
Billroth II	38 (21.2%)	198 (21.3%)		38 (21.2%)	78 (21.8%)	
Roux-en-Y	98 (54.7%)	516 (55.5%)		98 (54.7%)	192 (53.6%)	
Length of postoperative hospital stay (days)	5.9 ± 3.4	6.6 ± 5.2	0.03	5.9 ± 3.4	6.3 ± 5.1	0.23

Continuous variables are presented as the mean ± standard deviation

**Table 3:** Subgroup analysis of single-incision distal gastrectomy (SIDG) and multiport laparoscopic distal gastrectomy (MLDG) before and after 1:2 propensity score matching

		Before matching			After matching		
		SIDG (N = 112)	MLDG (N = 535)	*P* value	SIDG (N = 112)	MLDG (N = 235)	*P* value
25 ≤ BMI < 27.5	Operation time (min)	168.6 ± 64.8	186.8 ± 54.5	0.006	168.6 ± 64.8	182.6 ± 53.6	0.047
	Length of postoperative hospital stay (days)	6.0 ± 4.0	6.5 ± 4.5	0.25	6.0 ± 4.0	6.4 ± 5.4	0.38
	The number of patients with early postoperative complication	11 (9.8%)	57 (10.7%)	0.93	11 (9.8%)	28 (11.9%)	0.69
	Comprehensive Complication Index	2.0 ± 7.4	2.2 ± 7.0	0.75	2.0 ± 7.4	2.4 ± 7.4	0.57
	Highest Clavien-Dindo classification						
	I, II	8 (7.1%)	41 (7.7%)	> 0.99	8 (7.1%)	22 (9.4%)	0.63
	≥ IIIa	3 (2.7%)	16 (3.0%)	> 0.99	3 (2.7%)	6 (2.6%)	> 0.99

		Before matching			After matching		
		SIDG (N = 58)	MLDG (N = 275)	*P* value	SIDG (N = 58)	MLDG (N = 95)	*P* value
27.5 ≤ BMI < 30	Operation time (min)	171.9 ± 52.5	197.2 ± 52.5	0.001	171.9 ± 52.5	195.4 ± 52.0	0.008
	Length of postoperative hospital stay (days)	5.6 ± 2.0	6.2 ± 3.4	0.08	5.6 ± 2.0	5.8 ± 2.8	0.55
	The number of patients with early postoperative complication	11 (19.0%)	34 (12.4%)	0.26	11 (19.0%)	13 (13.7%)	0.52
	Comprehensive Complication Index	3.4 ± 7.8	2.1 ± 6.5	0.19	3.4 ± 7.8	2.4 ± 6.8	0.43
	Highest Clavien-Dindo classification						
	I, II	8 (13.8%)	26 (9.5%)	0.45	8 (13.8%)	9 (9.5%)	0.58
	≥ IIIa	3 (5.2%)	8 (2.9%)	0.64	3 (5.2%)	4 (4.2%)	> 0.99

		Before matching			After matching		
		SIDG (N = 9)	MLDG (N = 120)	*P* value	SIDG (N = 9)	MLDG (N = 28)	*P* value
BMI ≥ 30	Operation time (min)	191.1 ± 39.4	192.3 ± 53.1	0.95	191.1 ± 39.4	183.4 ± 43.8	0.64
	Length of postoperative hospital stay (days)	6.8 ± 2.5	7.8 ± 9.4	0.40	6.8 ± 2.5	7.2 ± 7.7	0.80
	The number of patients with early postoperative complication	2 (22.2%)	19 (15.8%)	0.64	2 (22.2%)	5 (17.9%)	> 0.99
	Comprehensive Complication Index	5.2 ± 10.5	3.2 ± 8.0	0.46	5.2 ± 10.5	3.0 ± 7.3	0.49
	Highest Clavien-Dindo classification						
	I, II	1 (11.1%)	15 (12.5%)	> 0.99	1 (11.1%)	4 (14.3%)	> 0.99
	≥ IIIa	1 (11.1%)	4 (3.3%)	0.31	1 (11.1%)	1 (3.6%)	0.43

As shown in Table 4, there was no significant difference in the early postoperative complication rate between the two groups before (SIDG group: 13.4%, MLDG group: 11.8%, *P* = 0.53) or after PSM (SIDG group: 13.4%, MLDG group: 12.8%, *P* = 0.89). Similarly, no significant differences were seen in the CCI between the two groups before (SIDG group: 2.6 ± 7.7 vs. MLDG group: 2.3 ± 7.0, *P* = 0.61) or after PSM (SIDG group: 2.6 ± 7.7 vs. MLDG group: 2.5 ± 7.2, *P* = 0.88). The distribution of individuals with Clavien-Dindo classification grade IIIa or higher did not differ between the two groups (SIDG group: 3.9% vs. MLDG group: 3.0%, *P* = 0.49). No cases of early postoperative mortality occurred in either group, and the incidence of local complications was not different after PSM (SIDG group: 6.7%, MLDG group: 6.1%, *P* = 0.74). The most common local complication was motility disorder in both groups (SIDG group: 2.2%, MLDG group: 2.2%). In the SIDG group, the most common local complication was anastomosis stricture in the BMI 25–27.5 kg/m^2^ subgroup (2.7%), motility disorder in the BMI 27.5–30 kg/m^2^ subgroup (3.4%), and fluid collection in the BMI ≥ 30 kg/m^2^ subgroup (11.1%) (Supplementary Table 3). No differences were seen in systemic complications between the two groups (SIDG group: 6.1%, MLDG group: 6.7%, *P* = 0.86). Pulmonary complications were the most common systemic complication in the two groups. (SIDG group: 6.1%, MLDG group: 5.6%). This trend was maintained in the subgroup analysis according to BMI (Supplementary Table 3).

**Table 4:** Postoperative morbidity and mortality within 1 month in single-incision distal gastrectomy (SIDG) and multiport laparoscopic distal gastrectomy (MLDG) before and after 1:2 propensity score matching

	Before matching			After matching		
	SIDG (N = 179)	MLDG (N = 930)	*P* value	SIDG (N = 179)	MLDG (N = 358)	*P* value
The number of patients with early postoperative complication	24 (13.4%)	110 (11.8%)	0.53	24 (13.4%)	46 (12.8%)	0.89
Comprehensive Complication Index	2.6 ± 7.7	2.3 ± 7.0	0.61	2.6 ± 7.7	2.5 ± 7.2	0.88
Highest Clavien-Dindo Classification			0.73			0.91
I	9 (5.0%)	37 (4.0%)	0.54	9 (5.0%)	15 (4.2%)	0.66
II	8 (4.5%)	45 (4.8%)	> 0.99	8 (4.5%)	20 (5.6%)	0.68
IIIa	6 (3.4%)	25 (2.7%)	0.62	6 (3.4%)	9 (2.5%)	0.59
IIIb	0 (0.0%)	1 (0.1%)	> 0.99	0 (0.0%)	1 (0.3%)	> 0.99
IVa	1 (0.6%)	2 (0.2%)	0.41	1 (0.6%)	1 (0.3%)	> 0.99
IVb	0 (0.0%)	0 (0.0%)	> 0.99	0 (0.0%)	0 (0.0%)	> 0.99
V	0 (0.0%)	0 (0.0%)	> 0.99	0 (0.0%)	0 (0.0%)	> 0.99
Local complication	12 (6.7%)	57 (6.1%)	0.74	12 (6.7%)	22 (6.1%)	0.85
Wound	1 (0.6%)	3 (0.3%)	0.51	1 (0.6%)	1 (0.3%)	> 0.99
Fluid collection	2 (1.1%)	10 (1.1%)	> 0.99	2 (1.1%)	5 (1.4%)	> 0.99
Intra-abdominal bleeding	1 (0.6%)	3 (0.3%)	0.51	1 (0.6%)	2 (0.6%)	> 0.99
Intra-luminal bleeding	0 (0.0%)	3 (0.3%)	> 0.99	0 (0.0%)	0 (0.0%)	> 0.99
Motility disorder	4 (2.2%)	18 (1.9%)	0.77	4 (2.2%)	8 (2.2%)	> 0.99
Anastomosis stricture	3 (1.7%)	6 (0.6%)	0.17	3 (1.7%)	1 (0.3%)	0.11
Anastomosis leakage	1 (0.6%)	8 (0.9%)	> 0.99	1 (0.6%)	4 (1.1%)	0.67
Stump leakage	1 (0.6%)	5 (0.5%)	> 0.99	1 (0.6%)	1 (0.3%)	> 0.99
Pancreatitis	1 (0.6%)	2 (0.2%)	0.41	1 (0.6%)	1 (0.3%)	> 0.99
Systemic complication	11 (6.1%)	58 (6.2%)	> 0.99	11 (6.1%)	24 (6.7%)	0.86
Pulmonary	11 (6.1%)	46 (4.9%)	0.46	11 (6.1%)	20 (5.6%)	0.85
Urinary	0 (0.0%)	3 (0.3%)	> 0.99	0 (0.0%)	2 (0.6%)	0.56
Renal	0 (0.0%)	1 (0.1%)	> 0.99	0 (0.0%)	1 (0.3%)	> 0.99
Gastrointestinal	0 (0.0%)	1 (0.1%)	> 0.99	0 (0.0%)	1 (0.3%)	> 0.99
Hepatobiliary	0 (0.0%)	5 (0.5%)	> 0.99	0 (0.0%)	1 (0.3%)	> 0.99
Neuropsychiatric	0 (0.0%)	3 (0.3%)	> 0.99	0 (0.0%)	0 (0.0%)	> 0.99
Cardiac	0 (0.0%)	1 (0.1%)	> 0.99	0 (0.0%)	0 (0.0%)	> 0.99
Vascular	0 (0.0%)	2 (0.2%)	> 0.99	0 (0.0%)	0 (0.0%)	> 0.99
Endocrine	0 (0.0%)	0 (0.0%)	> 0.99	0 (0.0%)	0 (0.0%)	> 0.99
Others complication^a^	1 (0.6%)	4 (0.4%)	0.59	1 (0.6%)	2 (0.6%)	> 0.99

Continuous variables are presented as the mean ± standard deviation

In the case of complication, it is expressed as the number of patients (%)

^a^Other complications: fever of unknown origin (5), all Clavien-Dindo grade II

**Fig. 3:**
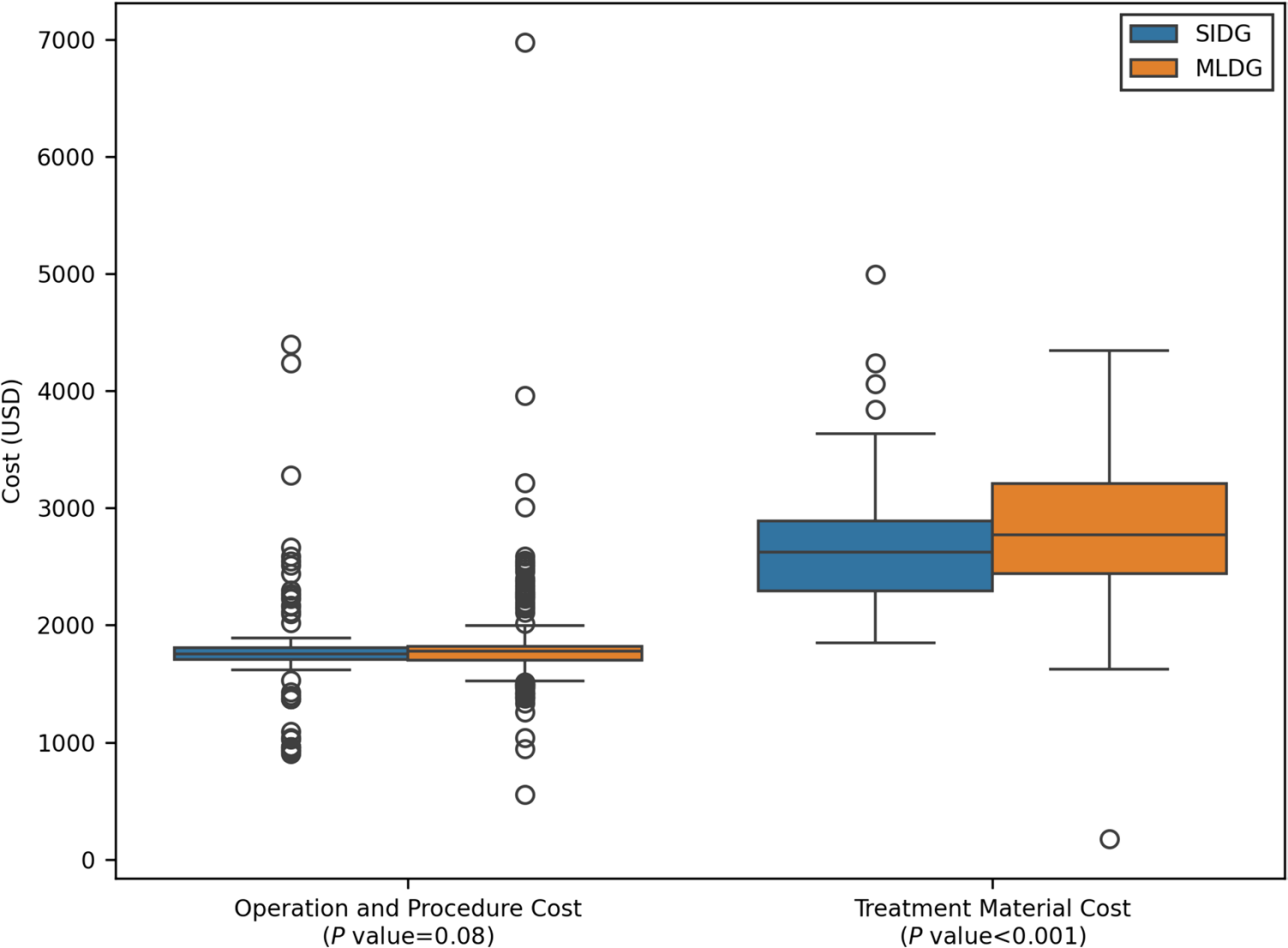
Comparison of operation and procedure cost, and treatment material cost between single-incision distal gastrectomy (SIDG) and multiport laparoscopic distal gastrectomy (MLDG) groups

The median costs of different categories were compared between the SIDG and MLDG groups. For total hospital cost, the SIDG group had a median cost of $7556 (IQR 6879, 8457), while the MLDG group had a median cost of $7601 (IQR 6988, 8320) (*P* = 0.46). In terms of operation and procedure cost, the SIDG group had a median cost of $1754 (IQR 1708, 1809), while the MLDG group had a median cost of $1777 (IQR 1700, 1820) (*P* = 0.08). Regarding treatment material cost, the SIDG group had a median cost of $2621 (IQR 2295, 2891), while the MLDG group had a significantly higher median cost of $2774 (IQR 2439, 3209) (*P* < 0.001) (Fig. 3). Data from the Korean Health Insurance Review and Assessment Service indicate that the indirect cost for the assistant surgeon’s workload for distal gastrectomy with LND is approximately 279,280 KRW (202 USD).

In the subgroup analysis (Supplementary Table 3) of individuals with a BMI between 25 and 27.5 kg/m^2^, a difference in the occurrence of anastomosis strictures was found between the SIDG group (three cases, 2.7%) and the MLDG group (0 cases, 0.0%) after 1:2 PSM (*P* = 0.03). Specifically, within the SIDG group, three cases of anastomosis strictures occurred: a 78-year-old female, who underwent Billroth I anastomosis, developed an anastomosis stricture resulting in aspiration pneumonia requiring intensive care unit management (Clavien-Dindo grade IVa). This patient underwent conversion surgery to gastrojejunostomy after conservative management failed. A 60-year-old male, who had Billroth I anastomosis, experienced an anastomosis stricture and was treated with balloon dilatation (Clavien-Dindo grade IIIa). Lastly, a 47-year-old female, who underwent Roux-en-Y gastrojejunostomy, was discharged following conservative management for the anastomosis stricture (Clavien-Dindo grade II).

The results of the subgroup analysis after 1:2 PSM by sex are shown in Supplementary Table 4. The operation time was shorter in the SIDG group than in the MLDG group in both males and females (males; SIDG group: 176.4 ± 63.5 vs. MLDG group: 191.4 ± 51.8,* P* = 0.049; females: SIDG group: 164.7 ± 55.6 vs. MLDG group: 179.8 ± 53.1, *P* = 0.04) (Supplementary Table 5). The hospital length of stay after surgery tended to be shorter in the SIDG group than in the MLDG group in both males and females, but there was no statistical difference (males: SIDG group: 5.7 ± 2.0 vs. MLDG group: 6.4 ± 5.0, *P* = 0.12; females: SIDG group: 6.1 ± 4.5 vs. MLDG group: 6.3 ± 5.3, *P* = 0.73). No differences were seen in complication rates, CCI values, local complication rates, and systemic complication rates between males and females in the SIDG and MLDG groups (Supplementary Table 6).

## Discussion

In this study, we critically assessed the safety and feasibility of SIDG in overweight and obese patients with gastric cancer. The evidence for the possible advantages of single-port gastrectomy is still accumulating [7–10]. Single-port surgery is technically challenging, and there is an inevitable limitation in creating a surgical field of view. Previous studies speculated that SIDG would be more challenging in obese patients due to the difficulty in creating an adequate surgical field of view compared to conventional multiport surgery [15]. However, to the best of our knowledge, our study was the first to specifically evaluate the safety of this single-port approach in an obese population. In our investigation, SIDG was evaluated as a secure procedure for early-stage gastric cancer patients with obesity. Compared to conventional multiport gastrectomy, an unexpectedly favorable outcome was noted in operation time as experience with this procedure accumulated. Several factors could have contributed to the efficacy of single-port gastrectomy over conventional multiport gastrectomy. One contributing factor could be the omission of the need for coordination between the surgeon and a trainee assistant during SIDG when compared to the coordination required in conventional multiport gastrectomy. MLDG is a highly assistant-dependent procedure, varying with the assistant’s familiarity with the procedure. In contrast, each procedure of SIDG has been developed to be standardized with minimal assistance. In our institution, the learning curve for SIDG has been analyzed to be approximately 30 cases. After this number of cases, similar results can be expected even in obese gastric cancer patients. The recent introduction of advanced surgical instruments, such as intracorporeal self-retractors and articulating devices has made SIDG more feasible for additional steady assistance when needed. Self-intracorporeal retractors could create a surgical environment that closely resembles the traction provided by an assistant in conventional multiport gastrectomy, and articulating instruments facilitate access to difficult suprapancreatic LND. These devices are expected to play a more crucial role in D2 LND for advanced gastric cancer (AGC). Another possible explanation is that during single-port gastrectomy, the approach to the infrapyloric lymph node station, which is a crucial aspect of distal gastrectomy, is more effective in the umbilical approach used in the SIDG group than the right-side approach used in the MLDG group.

A cost analysis comparing SIDG and MLDG showed no significant differences in total hospital cost or operation and procedure cost. However, the treatment material cost was significantly lower for SIDG. Although the Korean National Health Insurance Service does not vary the surgical fee based on an assistant surgeon’s participation, considering that the labor cost of the assistant surgeon is indirectly calculated to be 202 USD, SIDG can still be said to save this money indirectly. From a cosmetic perspective, numerous studies have reported the superiority of single-incision laparoscopic surgery (SILS). Specifically, SIDG has been demonstrated to be superior in terms of cosmesis, leaving only a single scar at the umbilicus [15, 27]. However, the issue of trainee assistant education in single-port gastrectomy remains an important challenge that must be addressed in the future.

In our study, patients visited the outpatient clinic to see their chosen surgeons without knowing the surgeon’s preference for SIDG or MLDG. The surgeons then determined the surgical method based on their preference. This approach led to a selection of SIDG or MLDG that closely resembled random allocation, as patients were not assigned specifically to SIDG or MLDG. One of the key strengths of this study is the robust comparison framework established between the SIDG and MLDG groups. Notably, the control group undergoing MLDG included more experienced physicians, providing a reliable basis for comparison and making our results more reliable and conservative.

Additionally, acknowledging the relative recency of SIDG, our methodology included PSM to reduce potential biases, particularly those arising from differences in the timing of surgery. We incorporated the year of surgery as a covariate in our analysis. This strategy was aimed at ensuring a similar distribution of the year of surgery for both groups, as evidenced in Table 1. Such an approach was critical for effectively minimizing any potential bias that might have arisen from variations in the year of surgery, further strengthening the validity of our comparisons. The results of this study demonstrated that the SIDG and MLDG procedures were comparable not only in terms of early postoperative complications but also in the number of retrieved lymph nodes, which implies the fundamental oncologic safety of SIDG.

In the study, the pathologic N stage was used as a matching variable. Due to the limitations of current diagnostic modalities, accurately assessing the clinical N stage preoperatively is challenging. Clinical N staging has low sensitivity and may introduce bias in PSM [28]. Importantly, metastatic lymph nodes significantly impact surgery compared to non-metastatic ones. If the number of retrieved lymph nodes is sufficiently similar between groups, pathologic LN staging is not influenced by surgery itself, but only reflects the preoperative status. Therefore, we considered pathologic N staging a reliable surrogate marker for preoperative status and reasonable for use as a matching variable. Statistical advice on the PSM variables was provided to us by the Medical Research Collaborating Center of our institution.

In our study, which primarily included EGC patients undergoing D1 + LND, we found that D2 LND was more frequently performed in the SIDG group, reflecting a cautious approach to new surgical techniques (SIDG: 39.1% vs. MLDG: 18.7%, *P* < 0.001). Subgroup analysis showed no significant difference in early postoperative complications, including pancreatic fistula, between SIDG and MLDG, regardless of the extent of LND.

We performed subgroup analysis based on gender to investigate whether any differences between the SIDG and MLDG groups could be attributed to sex-based distinctions. It is generally known that men have higher amounts of visceral fat, while women typically have a greater proportion of subcutaneous fat [29, 30]. No significant differences were observed in the comparison of postoperative hospital stays and overall complication rates between male and female patients in the SIDG and MLDG groups.

Recent studies have shown that robotic gastrectomy offers advantages over conventional laparoscopic surgery for obese gastric cancer patients, including shorter hospital stays and faster recovery [31]. The efficacy of reduced port gastrectomy, particularly in distal gastrectomy for EGC, has also been demonstrated [32]. Although the current Da Vinci SP System lacks essential instruments for gastrectomy, including robotic energy devices and linear staplers, limiting its potential to fully replace SILS, combining the SIDG technique with robotic surgery holds promise for improving outcomes in obese patients in the future.

A potential limitation of this study was the inherent selection bias due to differences in physician preferences and criteria for choosing between SIDG and MLDG. Although this bias may have persisted, the choice was dependent on patients’ random visits to the outpatient clinic. Despite our methodological efforts, this bias may still have been present, as surgeon allocation to either procedure was not random. Another limitation is the lack of data on the frequency of additional port placement due to the study’s retrospective nature. Surgical records were insufficient to determine whether reduced port surgery (two or three ports) initially started or if additional ports were added during SIDG. Therefore, we excluded cases with reduced ports and compared only SIDG and MLDG. Additionally, biases could have been introduced by the timing of the surgical procedure, particularly since SIDG is a more recently introduced procedure compared to MLDG, and this temporal difference could have impacted the results. Furthermore, the prevalence of patients with a BMI ≥ 30 kg/m^2^ is lower in Korea than in Western countries, limiting the generalizability of our findings to Western countries. Lastly, the short-term follow-up period limits our understanding of long-term survival outcomes. Nevertheless, this study evaluated oncologic safety between SIDG and MLDG in obese gastric cancer patients by comparing early postoperative complications, the number of harvested lymph nodes, and safety resection margin, which are the most powerful predictive markers for the long-term prognosis of EGC. Previous studies, including retrospective analyses and a randomized controlled trial, found comparable long-term outcomes between SIDG and MLDG [7, 9, 10]. Because the long-term safety and efficacy of SIDG in obese patients remain uncertain, comprehensive long-term follow-up studies on survival and recurrence are required to establish more definite evidence regarding oncologic safety.

A study investigated the safety and feasibility of SIDG in obese patients with pathologic EGC. The results provide a foundation for implementing SIDG in both EGC and obese AGC patients. Our institution is conducting the SPACE-01 trial (ClinicalTrials.gov Identifier: NCT05076279), a phase 2 trial to verify the safety and efficacy of single or reduced ports laparoscopic gastrectomy for AGC.

This study demonstrated the safety of SIDG in obese patients regarding early postoperative complications and oncological outcomes without compromising operation time. The safety of this surgical technique in obese patients provides a basis for phase 3 clinical trials in the future. Following this result, we also highlight the necessity for further investigation into the impact of SIDG on postoperative pain and quality of life in those patients.

In conclusion, SIDG is feasible and safe for overweight and obese gastric cancer patients with comparable early postoperative complication rates without compromising operation time compared to MLDG.

### Supplementary Information

Below is the link to the electronic supplementary material.

Supplementary file1 (DOCX 127 KB)
