# Supplementary material for: Safety and feasibility of single-incision laparoscopic distal gastrectomy in overweight and obese gastric cancer patients: a propensity score-matched analysis
Source: Gastric Cancer. 2024 Jul 18;27(5):1136–46. doi: 10.1007/s10120-024-01530-5 (PMC11335889; doi:10.1007/s10120-024-01530-5)
Supplement: Supplementary file 1 — Supplementary file1 (DOCX 127 KB) [file 10120_2024_1530_MOESM1_ESM.docx]

Supplementary Table 1. Clinicopathologic characteristics in body mass index (BMI) subgroups for single-incision distal gastrectomy (SIDG) and multiport laparoscopic distal gastrectomy (MLDG) before and after 1:2 propensity score matching

| 25 ≤ BMI < 27.5 |  | Before matching | | | After matching | | | |
| --- | --- | --- | --- | --- | --- | --- | --- | --- |
|  |  | SIDG  (N=112) | MLDG  (N=535) | *P* value | SIDG  (N=112) | MLDG  (N=235) | *P* value |  |
|  | Sex (M:F) | 66:46  (Male 58.9%) | 406:129  (Male 75.9%) | < 0.001 | 66:46  (Male 58.9%) | 139:96  (Male 59.1%) | >0.99 |  |
|  | Age (years) | 61.0 ± 11.6 | 60.9 ± 11.1 | 0.90 | 61.0 ± 11.6 | 61.4 ± 11.7 | 0.80 |  |
|  | Height (cm) | 161.8 ± 8.9 | 164.9 ± 8.6 | 0.001 | 161.8 ± 8.9 | 161.5 ± 9.3 | 0.81 |  |
|  | Weight (kg) | 68.6 ± 8.0 | 71.2 ± 7.7 | 0.002 | 68.6 ± 8.0 | 68.0 ± 8.0 | 0.47 |  |
|  | BMI (kg/m^2^) | 26.1 ± 0.8 | 26.1 ± 0.7 | 0.59 | 26.1 ± 0.8 | 26.0 ± 0.7 | 0.59 |  |
|  | ASA^[[1]](#footnote-1)^ classification |  |  | 0.79 |  |  | 0.78 |  |
|  | I | 35 (31.2%) | 156 (29.2%) |  | 35 (31.2%) | 70 (29.8%) |  |  |
|  | II | 70 (62.5%) | 351 (65.6%) |  | 70 (62.5%) | 150 (63.8%) |  |  |
|  | III | 7 (6.2%) | 26 (4.9%) |  | 7 (6.2%) | 13 (5.5%) |  |  |
|  | IV | 0 (0.0%) | 2 (0.4%) |  | 0 (0.0%) | 2 (0.9%) |  |  |
|  | Year of operation |  |  | 0.001 |  |  | 0.70 |  |
|  | 2011 | 0 (0.0%) | 6 (1.1%) |  | 0 (0.0%) | 0 (0.0%) |  |  |
|  | 2012 | 2 (1.8%) | 19 (3.6%) |  | 2 (1.8%) | 2 (0.9%) |  |  |
|  | 2013 | 3 (2.7%) | 37 (6.9%) |  | 3 (2.7%) | 13 (5.5%) |  |  |
|  | 2014 | 5 (4.5%) | 76 (14.2%) |  | 5 (4.5%) | 14 (6.0%) |  |  |
|  | 2015 | 18 (16.1%) | 76 (14.2%) |  | 18 (16.1%) | 34 (14.5%) |  |  |
|  | 2016 | 20 (17.9%) | 96 (17.9%) |  | 20 (17.9%) | 34 (14.5%) |  |  |
|  | 2017 | 20 (17.9%) | 82 (15.3%) |  | 20 (17.9%) | 35 (14.9%) |  |  |
|  | 2018 | 10 (8.9%) | 59 (11.0%) |  | 10 (8.9%) | 33 (14.0%) |  |  |
|  | 2019 | 13 (11.6%) | 46 (8.6%) |  | 13 (11.6%) | 34 (14.5%) |  |  |
|  | 2020 | 14 (12.5%) | 29 (5.4%) |  | 14 (12.5%) | 27 (11.5%) |  |  |
|  | 2021 | 7 (6.2%) | 9 (1.7%) |  | 7 (6.2%) | 9 (3.8%) |  |  |
|  | Tumor size (cm) | 2.8 ± 1.7 | 2.5 ± 1.6 | 0.18 | 2.8 ± 1.7 | 2.7 ± 1.7 | 0.80 |  |
|  | Proximal resection margin (cm) | 4.6 ± 2.8 | 4.5 ± 2.8 | 0.71 | 4.6 ± 2.8 | 4.4 ± 2.7 | 0.38 |  |
|  | Distal resection margin (cm) | 5.2 ± 3.3 | 5.4 ± 2.8 | 0.45 | 5.2 ± 3.3 | 5.2 ± 2.7 | 0.91 |  |
|  | Number of retrieved lymph nodes | 52.7 ± 19.2 | 54.2 ± 21.4 | 0.50 | 52.7 ± 19.2 | 52.8 ± 19.8 | 0.98 |  |
|  | Number of positive lymph nodes | 0.3 ± 0.8 | 0.3 ± 1.1 | 0.75 | 0.3 ± 0.8 | 0.4 ± 1.2 | 0.20 |  |
|  | Lymphatic invasion | 21 (18.8%) | 65 (12.2%) | 0.09 | 21 (18.8%) | 35 (14.9%) | 0.45 |  |
|  | Venous invasion | 1 (0.9%) | 6 (1.1%) | >0.99 | 1 (0.9%) | 3 (1.3%) | >0.99 |  |
|  | Perineural invasion | 8 (7.1%) | 22 (4.2%) | 0.26 | 8 (7.1%) | 11 (4.7%) | 0.50 |  |
|  | Pathologic T stage |  |  | 0.38 |  |  | 0.79 |  |
|  | pT1a | 59 (52.7%) | 309 (57.8%) |  | 59 (52.7%) | 129 (54.9%) |  |  |
|  | pT1b | 53 (47.3%) | 226 (42.2%) |  | 53 (47.3%) | 106 (45.1%) |  |  |
|  | Pathologic N stage |  |  | 0.13 |  |  | 0.50 |  |
|  | pN0 | 96 (85.7%) | 478 (89.3%) |  | 96 (85.7%) | 197 (83.8%) |  |  |
|  | pN1 | 13 (11.6%) | 32 (6.0%) |  | 13 (11.6%) | 24 (10.2%) |  |  |
|  | pN2 | 3 (2.7%) | 20 (3.7%) |  | 3 (2.7%) | 11 (4.7%) |  |  |
|  | pN3a | 0 (0.0%) | 5 (0.9%) |  | 0 (0.0%) | 3 (1.3%) |  |  |
|  | Pathologic TNM stage |  |  | 0.16 |  |  | >0.99 |  |
|  | Stage I | 97 (86.6%) | 489 (91.4%) |  | 97 (86.6%) | 205 (87.2%) |  |  |
|  | Stage II | 15 (13.4%) | 46 (8.6%) |  | 15 (13.4%) | 30 (12.8%) |  |  |
| 27.5 ≤ BMI < 30 |  | Before matching | | | After matching | | | |
|  |  | SIDG  (N=58) | MLDG  (N=275) | *P* value | SIDG  (N=58) | MLDG  (N=95) | *P* value |  |
|  | Sex (M:F) | 24:34  (Male 41.4%) | 196:79  (Male 71.3%) | <0.001 | 24:34  (Male 41.4%) | 47:48  (Male 49.5%) | 0.42 |  |
|  | Age (years) | 63.4 ± 11.4 | 61.4 ± 10.6 | 0.18 | 63.4 ± 11.4 | 62.9 ± 9.7 | 0.74 |  |
|  | Height (cm) | 159.5 ± 8.2 | 163.6 ± 9.1 | 0.002 | 159.5 ± 8.2 | 159.9 ± 9.0 | 0.79 |  |
|  | Weight (kg) | 72.4 ± 7.4 | 76.5 ± 8.6 | 0.001 | 72.4 ± 7.4 | 72.9 ± 8.3 | 0.76 |  |
|  | BMI (kg/m^2^) |  |  |  | 28.4 ± 0.7 | 28.4 ± 0.7 | 0.98 |  |
|  | ASA classification |  |  | 0.52 |  |  | 0.64 |  |
|  | I | 17 (29.3%) | 71 (25.8%) |  | 17 (29.3%) | 27 (28.4%) |  |  |
|  | II | 35 (60.3%) | 185 (67.3%) |  | 35 (60.3%) | 62 (65.3%) |  |  |
|  | III | 6 (10.3%) | 19 (6.9%) |  | 6 (10.3%) | 6 (6.3%) |  |  |
|  | IV | 0 (0.0%) | 0 (0.0%) |  | 0 (0.0%) | 0 (0.0%) |  |  |
|  | Year of operation |  |  | 0.19 |  |  | 0.36 |  |
|  | 2011 | 0 (0.0%) | 4 (1.5%) |  |  |  |  |  |
|  | 2012 | 1 (1.7%) | 9 (3.3%) |  | 1 (1.7%) | 3 (3.2%) |  |  |
|  | 2013 | 3 (5.2%) | 19 (6.9%) |  | 3 (5.2%) | 7 (7.4%) |  |  |
|  | 2014 | 3 (5.2%) | 36 (13.1%) |  | 3 (5.2%) | 0 (0.0%) |  |  |
|  | 2015 | 5 (8.6%) | 45 (16.4%) |  | 5 (8.6%) | 16 (16.8%) |  |  |
|  | 2016 | 9 (15.5%) | 48 (17.5%) |  | 9 (15.5%) | 20 (21.1%) |  |  |
|  | 2017 | 9 (15.5%) | 37 (13.5%) |  | 9 (15.5%) | 12 (12.6%) |  |  |
|  | 2018 | 8 (13.8%) | 29 (10.5%) |  | 8 (13.8%) | 10 (10.5%) |  |  |
|  | 2019 | 7 (12.1%) | 15 (5.5%) |  | 7 (12.1%) | 6 (6.3%) |  |  |
|  | 2020 | 11 (19.0%) | 28 (10.2%) |  | 11 (19.0%) | 17 (17.9%) |  |  |
|  | 2021 | 2 (3.4%) | 5 (1.8%) |  | 2 (3.4%) | 4 (4.2%) |  |  |
|  | Tumor size (cm) | 2.5 ± 1.3 | 2.6 ± 1.5 | 0.81 | 2.5 ± 1.3 | 2.6 ± 1.7 | 0.61 |  |
|  | Proximal resection margin (cm) | 4.7 ± 2.6 | 4.9 ± 3.1 | 0.68 | 4.7 ± 2.6 | 4.9 ± 3.1 | 0.71 |  |
|  | Distal resection margin (cm) | 5.1 ± 3.1 | 5.5 ± 3.1 | 0.44 | 5.1 ± 3.1 | 5.1 ± 2.7 | 0.97 |  |
|  | Number of retrieved lymph nodes | 53.0 ± 20.5 | 56.3 ± 22.2 | 0.30 | 53.0 ± 20.5 | 56.8 ± 23.6 | 0.31 |  |
|  | Number of positive lymph nodes | 0.7 ± 1.7 | 0.3 ± 1.2 | 0.09 | 0.7 ± 1.7 | 0.4 ± 1.5 | 0.38 |  |
|  | Lymphatic invasion | 9 (15.5%) | 40 (14.5%) | >0.99 | 9 (15.5%) | 18 (18.9%) | 0.75 |  |
|  | Venous invasion | 0 (0.0%) | 6 (2.2%) | 0.55 | 0 (0.0%) | 3 (3.2%) | 0.44 |  |
|  | Perineural invasion | 1 (1.7%) | 7 (2.6%) | >0.99 | 1 (1.7%) | 1 (1.1%) | >0.99 |  |
|  | Pathologic T stage |  |  | 0.44 |  |  | 0.95 |  |
|  | pT1a | 31 (53.4%) | 165 (60.0%) |  | 31 (53.4%) | 49 (51.6%) |  |  |
|  | pT1b | 27 (46.6%) | 110 (40.0%) |  | 27 (46.6%) | 46 (48.4%) |  |  |
|  | Pathologic N stage |  |  | 0.05 |  |  | 0.68 |  |
|  | pN0 | 45 (77.6%) | 247 (89.8%) |  | 45 (77.6%) | 77 (81.1%) |  |  |
|  | pN1 | 8 (13.8%) | 19 (6.9%) |  | 8 (13.8%) | 14 (14.7%) |  |  |
|  | pN2 | 3 (5.2%) | 7 (2.5%) |  | 3 (5.2%) | 3 (3.2%) |  |  |
|  | pN3a | 2 (3.4%) | 2 (0.7%) |  | 2 (3.4%) | 1 (1.1%) |  |  |
|  | Pathologic TNM stage |  |  | 0.008 |  |  | 0.47 |  |
|  | Stage I | 46 (79.3%) | 253 (92.0%) |  | 46 (79.3%) | 81 (85.3%) |  |  |
|  | Stage II | 12 (20.7%) | 22 (8.0%) |  | 12 (20.7%) | 14 (14.7%) |  |  |
| BMI ≥ 30 |  | Before matching | | | After matching | | | |
|  |  | SIDG  (N=9) | MLDG  (N=120) | *P* value | SIDG  (N=9) | MLDG  (N=28) | *P* value |  |
|  | Sex (M:F) | 3:6  (Male 33.3%) | 66:54  (Male 55.0%) | 0.36 | 3:6  (Male 33.3%) | 8:20 (Male 28.6%) | >0.99 |  |
|  | Age (years) | 61.6 ± 7.9 | 59.1 ± 12.9 | 0.57 | 61.6 ± 7.9 | 58.6 ± 13.8 | 0.54 |  |
|  | Height (cm) | 157.9 ± 11.0 | 162.6 ± 9.8 | 0.17 | 157.9 ± 11.0 | 159.7 ± 11.1 | 0.67 |  |
|  | Weight (kg) | 80.5 ± 15.4 | 85.2 ± 11.2 | 0.24 | 80.5 ± 15.4 | 80.7 ± 11.4 | 0.96 |  |
|  | BMI (kg/m^2^) | 32.0 ± 2.0 | 32.2 ± 2.2 | 0.82 | 32.0 ± 2.0 | 31.5 ± 1.5 | 0.48 |  |
|  | ASA classification |  |  | 0.98 |  |  | 0.62 |  |
|  | I | 2 (22.2%) | 29 (24.2%) |  | 2 (22.2%) | 9 (32.1%) |  |  |
|  | II | 6 (66.7%) | 76 (63.3%) |  | 6 (66.7%) | 18 (64.3%) |  |  |
|  | III | 1 (11.1%) | 15 (12.5%) |  | 1 (11.1%) | 1 (3.6%) |  |  |
|  | IV | 0 (0.0%) | 0 (0.0%) |  | 0 (0.0%) | 0 (0.0%) |  |  |
|  | Year of operation |  |  | 0.02 |  |  | 0.29 |  |
|  | 2011 | 0 (0.0%) | 2 (1.7%) |  | 0 (0.0%) | 0 (0.0%) |  |  |
|  | 2012 | 0 (0.0%) | 3 (2.5%) |  | 0 (0.0%) | 0 (0.0%) |  |  |
|  | 2013 | 2 (22.2%) | 6 (5.0%) |  | 2 (22.2%) | 2 (7.1%) |  |  |
|  | 2014 | 0 (0.0%) | 13 (10.8%) |  | 0 (0.0%) | 0 (0.0%) |  |  |
|  | 2015 | 1 (11.1%) | 22 (18.3%) |  | 1 (11.1%) | 7 (25.0%) |  |  |
|  | 2016 | 1 (11.1%) | 26 (21.7%) |  | 1 (11.1%) | 4 (14.3%) |  |  |
|  | 2017 | 0 (0.0%) | 14 (11.7%) |  | 0 (0.0%) | 2 (7.1%) |  |  |
|  | 2018 | 2 (22.2%) | 14 (11.7%) |  | 2 (22.2%) | 2 (7.1%) |  |  |
|  | 2019 | 1 (11.1%) | 7 (5.8%) |  | 1 (11.1%) | 2 (7.1%) |  |  |
|  | 2020 | 1 (11.1%) | 13 (10.8%) |  | 1 (11.1%) | 9 (32.1%) |  |  |
|  | 2021 | 1 (11.1%) | 0 (0.0%) |  | 1 (11.1%) | 0 (0.0%) |  |  |
|  | Tumor size (cm) | 2.4 ± 0.9 | 2.5 ± 1.5 | 0.72 | 2.4 ± 0.9 | 2.7 ± 2.1 | 0.56 |  |
|  | Proximal resection margin (cm) | 5.2 ± 2.6 | 5.1 ± 2.8 | 0.92 | 5.2 ± 2.6 | 4.6 ± 2.2 | 0.47 |  |
|  | Distal resection margin (cm) | 5.1 ± 2.2 | 5.8 ± 3.4 | 0.56 | 5.1 ± 2.2 | 6.1 ± 3.8 | 0.45 |  |
|  | Number of retrieved lymph nodes | 52.2 ± 14.9 | 52.8 ± 23.5 | 0.94 | 52.2 ± 14.9 | 53.3 ± 21.4 | 0.89 |  |
|  | Number of positive lymph nodes | 0.4 ± 1.0 | 0.3 ± 1.1 | 0.63 | 0.4 ± 1.0 | 0.3 ± 0.8 | 0.62 |  |
|  | Lymphatic invasion | 1 (11.1%) | 13 (10.8%) | >0.99 | 1 (11.1%) | 7 (25.0%) | 0.68 |  |
|  | Venous invasion | 0 (0.0%) | 0 (0.0%) | >0.99 | 0 (0.0%) | 0 (0.0%) | >0.99 |  |
|  | Perineural invasion | 0 (0.0%) | 4 (3.3%) | >0.99 | 0 (0.0%) | 0 (0.0%) | >0.99 |  |
|  | Pathologic T stage |  |  | 0.72 |  |  | 0.51 |  |
|  | pT1a | 4 (44.4%) | 68 (56.7%) |  | 4 (44.4%) | 18 (64.3%) |  |  |
|  | pT1b | 5 (55.6%) | 52 (43.3%) |  | 5 (55.6%) | 10 (35.7%) |  |  |
|  | Pathologic N stage |  |  | 0.27 |  |  | 0.68 |  |
|  | pN0 | 7 (77.8%) | 109 (90.8%) |  | 7 (77.8%) | 24 (85.7%) |  |  |
|  | pN1 | 1 (11.1%) | 7 (5.8%) |  | 1 (11.1%) | 3 (10.7%) |  |  |
|  | pN2 | 1 (11.1%) | 2 (1.7%) |  | 1 (11.1%) | 1 (3.6%) |  |  |
|  | pN3a | 0 (0.0%) | 2 (1.7%) |  | 0 (0.0%) | 0 (0.0%) |  |  |
|  | Pathologic TNM stage |  |  | >0.99 |  |  | >0.99 |  |
|  | Stage I | 8 (88.9%) | 111 (92.5%) |  | 8 (88.9%) | 26 (92.9%) |  |  |
|  | Stage II | 1 (11.1%) | 9 (7.5%) |  | 1 (11.1%) | 2 (7.1%) |  |  |

Continuous variables are presented as the mean ± standard deviation.

Supplementary Table 2. Surgical outcomes and postoperative course of single-incision distal gastrectomy (SIDG) and multiport laparoscopic distal gastrectomy (MLDG) by body mass index (BMI) subgroup before and after 1:2 propensity score matching

| 25 ≤ BMI < 27.5 |  | Before matching | | | After matching | | |
| --- | --- | --- | --- | --- | --- | --- | --- |
|  |  | SIDG  (N=112) | MLDG  (N=535) | *P* value | SIDG  (N=112) | MLDG  (N=235) | *P* value |
|  | Operation time (minutes) | 168.6 ± 64.8 | 186.8 ± 54.5 | 0.006 | 168.6 ± 64.8 | 182.6 ± 53.6 | 0.047 |
|  | Intraoperative transfusion | 0 (0.0%) | 2 (0.4%) | >0.99 | 0 (0.0%) | 1 (0.4%) | >0.99 |
|  | Concomitant prophylactic cholecystectomy and appendectomy | 4 (3.6%) | 12 (2.2%) | 0.63 | 4 (3.6%) | 7 (3.0%) | >0.99 |
|  | Anastomosis |  |  | 0.84 |  |  | 0.68 |
|  | Billroth I | 26 (23.2%) | 129 (24.1%) |  | 26 (23.2%) | 56 (23.8%) |  |
|  | Billroth II | 22 (19.6%) | 116 (21.7%) |  | 22 (19.6%) | 55 (23.4%) |  |
|  | Roux-en-Y | 64 (57.1%) | 290 (54.2%) |  | 64 (57.1%) | 124 (52.8%) |  |
|  | Length of postoperative hospital stay (days) | 6.0 ± 4.0 | 6.5 ± 4.5 | 0.25 | 6.0 ± 4.0 | 6.4 ± 5.4 | 0.38 |
| 27.5 ≤ BMI < 30 |  | Before matching | | | After matching | | |
|  |  | SIDG  (N=58) | MLDG  (N=275) | *P* value | SIDG  (N=58) | MLDG  (N=95) | *P* value |
|  | Operation time (minutes) | 171.9 ± 52.5 | 197.2 ± 52.5 | 0.001 | 171.9 ± 52.5 | 195.4 ± 52.0 | 0.008 |
|  | Intraoperative transfusion | 0 (0.0%) | 0 (0.0%) | >0.99 | 0 (0.0%) | 0 (0.0%) | >0.99 |
|  | Concomitant prophylactic cholecystectomy and appendectomy | 1 (1.7%) | 10 (3.6%) | 0.74 | 1 (1.7%) | 2 (2.1%) | >0.99 |
|  | Anastomosis |  |  | 0.41 |  |  | 0.37 |
|  | Billroth I | 15 (25.9%) | 66 (24.0%) |  | 15 (25.9%) | 27 (28.4%) |  |
|  | Billroth II | 16 (27.6%) | 57 (20.7%) |  | 16 (27.6%) | 17 (17.9%) |  |
|  | Roux-en-Y | 27 (46.6%) | 152 (55.3%) |  | 27 (46.6%) | 51 (53.7%) |  |
|  | Length of postoperative hospital stay (days) | 5.6 ± 2.0 | 6.2 ± 3.4 | 0.08 | 5.6 ± 2.0 | 5.8 ± 2.8 | 0.55 |
| BMI ≥ 30 |  | Before matching | | | After matching | | |
|  |  | SIDG  (N=9) | MLDG  (N=120) | *P* value | SIDG  (N=9) | MLDG  (N=28) | *P* value |
|  | Operation time (minutes) | 191.1 ± 39.4 | 192.3 ± 53.1 | 0.95 | 191.1 ± 39.4 | 183.4 ± 43.8 | 0.64 |
|  | Intraoperative transfusion | 0 (0.0%) | 0 (0.0%) | >0.99 | 0 (0.0%) | 0 (0.0%) | >0.99 |
|  | Concomitant prophylactic cholecystectomy and appendectomy | 1 (11.1%) | 5 (4.2%) | 0.89 | 1 (11.1%) | 1 (3.6%) | 0.98 |
|  | Anastomosis |  |  | 0.31 |  |  | 0.32 |
|  | Billroth I | 2 (22.2%) | 21 (17.5%) |  | 2 (22.2%) | 5 (17.9%) |  |
|  | Billroth II | 0 (0.0%) | 25 (20.8%) |  | 0 (0.0%) | 6 (21.4%) |  |
|  | Roux-en-Y | 7 (77.8%) | 74 (61.7%) |  | 7 (77.8%) | 17 (60.7%) |  |
|  | Length of postoperative hospital stay (days) | 6.8 ± 2.5 | 7.8 ± 9.4 | 0.40 | 6.8 ± 2.5 | 7.2 ± 7.7 | 0.80 |

Continuous variables are presented as the mean ± standard deviation.

Supplementary Table 3. Postoperative morbidity and mortality within 1 month by body mass index (BMI) subgroup in single-incision distal gastrectomy (SIDG) and multiport laparoscopic distal gastrectomy (MLDG) before and after 1:2 propensity score matching

| 25 ≤ BMI < 27.5 |  | Before matching | | | After matching | | |
| --- | --- | --- | --- | --- | --- | --- | --- |
|  |  | SIDG  (N=112) | MLDG  (N=535) | *P* value | SIDG  (N=112) | MLDG  (N=235) | *P* value |
|  | The number of patients with early postoperative complication | 11 (9.8%) | 57 (10.7%) | 0.93 | 11 (9.8%) | 28 (11.9%) | 0.69 |
|  | Comprehensive Complication Index | 2.0 ± 7.4 | 2.2 ± 7.0 | 0.75 | 2.0 ± 7.4 | 2.4 ± 7.4 | 0.57 |
|  | Highest Clavien-Dindo Classification |  |  |  |  |  |  |
|  | I | 5 (4.5%) | 15 (2.8%) | 0.53 | 5 (4.5%) | 8 (3.4%) | 0.85 |
|  | II | 3 (2.7%) | 26 (4.9%) | 0.45 | 3 (2.7%) | 14 (6.0%) | 0.29 |
|  | IIIa | 2 (1.8%) | 14 (2.6%) | 0.86 | 2 (1.8%) | 4 (1.7%) | >0.99 |
|  | IIIb | 0 (0.0%) | 1 (0.2%) | >0.99 | 0 (0.0%) | 1 (0.4%) | >0.99 |
|  | IVa | 1 (0.9%) | 1 (0.2%) | 0.77 | 1 (0.9%) | 1 (0.4%) | >0.99 |
|  | IVb | 0 (0.0%) | 0 (0.0%) | >0.99 | 0 (0.0%) | 0 (0.0%) | >0.99 |
|  | V | 0 (0.0%) | 0 (0.0%) | >0.99 | 0 (0.0%) | 0 (0.0%) | >0.99 |
|  | Highest Clavien-Dindo Classification |  |  |  |  |  |  |
|  | I, II | 8 (7.1%) | 41 (7.7%) | >0.99 | 8 (7.1%) | 22 (9.4%) | 0.63 |
|  | ≥IIIa | 3 (2.7%) | 16 (3.0%) | >0.99 | 3 (2.7%) | 6 (2.6%) | >0.99 |
|  | **Local complication** | 7 (6.2%) | 29 (5.4%) | 0.66 | 7 (6.2%) | 12 (5.1%) | 0.62 |
|  | Wound | 1 (0.9%) | 1 (0.2%) | 0.32 | 1 (0.9%) | 0 (0.0%) | 0.32 |
|  | I, II | 1 (0.9%) | 0 (0.0%) | 0.17 | 1 (0.9%) | 0 (0.0%) | 0.32 |
|  | ≥IIIa | 0 (0.0%) | 1 (0.2%) | >0.99 | 0 (0.0%) | 0 (0.0%) | >0.99 |
|  | Fluid collection | 0 (0.0%) | 6 (1.1%) | 0.60 | 0 (0.0%) | 3 (1.3%) | 0.55 |
|  | I, II | 0 (0.0%) | 2 (0.4%) | >0.99 | 0 (0.0%) | 2 (0.9%) | >0.99 |
|  | ≥IIIa | 0 (0.0%) | 4 (0.7%) | >0.99 | 0 (0.0%) | 1 (0.4%) | >0.99 |
|  | Intra-abdominal bleeding | 0 (0.0%) | 1 (0.2%) | >0.99 | 0 (0.0%) | 1 (0.4%) | >0.99 |
|  | I, II | 0 (0.0%) | 0 (0.0%) | >0.99 | 0 (0.0%) | 0 (0.0%) | >0.99 |
|  | ≥IIIa | 0 (0.0%) | 1 (0.2%) | >0.99 | 0 (0.0%) | 1 (0.4%) | >0.99 |
|  | Intra-luminal bleeding | 0 (0.0%) | 1 (0.2%) | >0.99 | 0 (0.0%) | 0 (0.0%) | >0.99 |
|  | I, II | 0 (0.0%) | 1 (0.2%) | >0.99 | 0 (0.0%) | 0 (0.0%) | >0.99 |
|  | ≥IIIa | 0 (0.0%) | 0 (0.0%) | >0.99 | 0 (0.0%) | 0 (0.0%) | >0.99 |
|  | Motility disorder | 2 (1.8%) | 11 (2.1%) | >0.99 | 2 (1.8%) | 6 (2.6%) | >0.99 |
|  | I, II | 2 (1.8%) | 11 (2.1%) | >0.99 | 2 (1.8%) | 6 (2.6%) | >0.99 |
|  | ≥IIIa | 0 (0.0%) | 0 (0.0%) | >0.99 | 0 (0.0%) | 0 (0.0%) | >0.99 |
|  | Anastomosis stricture | 3 (2.7%) | 3 (0.6%) | 0.07 | 3 (2.7%) | 0 (0.0%) | 0.03 |
|  | I, II | 1 (0.9%) | 1 (0.2%) | 0.32 | 1 (0.9%) | 0 (0.0%) | 0.32 |
|  | ≥IIIa | 2 (1.8%) | 2 (0.4%) | 0.14 | 2 (1.8%) | 0 (0.0%) | 0.10 |
|  | Anastomosis leakage | 1 (0.9%) | 3 (0.6%) | 0.53 | 1 (0.9%) | 2 (0.9%) | >0.99 |
|  | I, II | 0 (0.0%) | 1 (0.2%) | >0.99 | 0 (0.0%) | 1 (0.4%) | >0.99 |
|  | ≥IIIa | 1 (0.9%) | 2 (0.4%) | 0.44 | 1 (0.9%) | 1 (0.4%) | 0.54 |
|  | Stump leakage | 0 (0.0%) | 4 (0.7%) | >0.99 | 0 (0.0%) | 1 (0.4%) | >0.99 |
|  | I, II | 0 (0.0%) | 1 (0.2%) | >0.99 | 0 (0.0%) | 1 (0.4%) | >0.99 |
|  | ≥IIIa | 0 (0.0%) | 3 (0.6%) | >0.99 | 0 (0.0%) | 0 (0.0%) | >0.99 |
|  | Pancreatitis | 1 (0.9%) | 0 (0.0%) | 0.17 | 1 (0.9%) | 0 (0.0%) | 0.32 |
|  | I, II | 1 (0.9%) | 0 (0.0%) | 0.17 | 1 (0.9%) | 0 (0.0%) | 0.32 |
|  | ≥IIIa | 0 (0.0%) | 0 (0.0%) | >0.99 | 0 (0.0%) | 0 (0.0%) | >0.99 |
|  | **Systemic complication** | 5 (4.5%) | 30 (5.6%) | 0.82 | 5 (4.5%) | 16 (6.8%) | 0.48 |
|  | Pulmonary | 5 (4.5%) | 26 (4.9%) | >0.99 | 5 (4.5%) | 13 (5.5%) | 0.80 |
|  | I, II | 4 (3.6%) | 24 (4.5%) | 0.80 | 4 (3.6%) | 12 (5.1%) | 0.60 |
|  | ≥IIIa | 1 (0.9%) | 2 (0.4%) | 0.44 | 1 (0.9%) | 1 (0.4%) | 0.54 |
|  | Urinary | 0 (0.0%) | 2 (0.4%) | >0.99 | 0 (0.0%) | 1 (0.4%) | >0.99 |
|  | I, II | 0 (0.0%) | 2 (0.4%) | >0.99 | 0 (0.0%) | 1 (0.4%) | >0.99 |
|  | ≥IIIa | 0 (0.0%) | 0 (0.0%) | >0.99 | 0 (0.0%) | 0 (0.0%) | >0.99 |
|  | Renal | 0 (0.0%) | 1 (0.2%) | >0.99 | 0 (0.0%) | 1 (0.4%) | >0.99 |
|  | I, II | 0 (0.0%) | 0 (0.0%) | >0.99 | 0 (0.0%) | 0 (0.0%) | >0.99 |
|  | ≥IIIa | 0 (0.0%) | 1 (0.2%) | >0.99 | 0 (0.0%) | 1 (0.4%) | >0.99 |
|  | Gastrointestinal | 0 (0.0%) | 1 (0.2%) | >0.99 | 0 (0.0%) | 1 (0.4%) | >0.99 |
|  | I, II | 0 (0.0%) | 1 (0.2%) | >0.99 | 0 (0.0%) | 1 (0.4%) | >0.99 |
|  | ≥IIIa | 0 (0.0%) | 0 (0.0%) | >0.99 | 0 (0.0%) | 0 (0.0%) | >0.99 |
|  | Hepatobiliary | 0 (0.0%) | 1 (0.2%) | >0.99 | 0 (0.0%) | 1 (0.4%) | >0.99 |
|  | I, II | 0 (0.0%) | 0 (0.0%) | >0.99 | 0 (0.0%) | 0 (0.0%) | >0.99 |
|  | ≥IIIa | 0 (0.0%) | 1 (0.2%) | >0.99 | 0 (0.0%) | 1 (0.4%) | >0.99 |
|  | Neuropsychiatric | 0 (0.0%) | 0 (0.0%) | >0.99 | 0 (0.0%) | 0 (0.0%) | >0.99 |
|  | I, II | 0 (0.0%) | 0 (0.0%) | >0.99 | 0 (0.0%) | 0 (0.0%) | >0.99 |
|  | ≥IIIa | 0 (0.0%) | 0 (0.0%) | >0.99 | 0 (0.0%) | 0 (0.0%) | >0.99 |
|  | Cardiac | 0 (0.0%) | 1 (0.2%) | >0.99 | 0 (0.0%) | 0 (0.0%) | >0.99 |
|  | I, II | 0 (0.0%) | 1 (0.2%) | >0.99 | 0 (0.0%) | 0 (0.0%) | >0.99 |
|  | ≥IIIa | 0 (0.0%) | 0 (0.0%) | >0.99 | 0 (0.0%) | 0 (0.0%) | >0.99 |
|  | Vascular | 0 (0.0%) | 0 (0.0%) | >0.99 | 0 (0.0%) | 0 (0.0%) | >0.99 |
|  | I, II | 0 (0.0%) | 0 (0.0%) | >0.99 | 0 (0.0%) | 0 (0.0%) | >0.99 |
|  | ≥IIIa | 0 (0.0%) | 0 (0.0%) | >0.99 | 0 (0.0%) | 0 (0.0%) | >0.99 |
|  | Endocrine | 0 (0.0%) | 0 (0.0%) | >0.99 | 0 (0.0%) | 0 (0.0%) | >0.99 |
|  | I, II | 0 (0.0%) | 0 (0.0%) | >0.99 | 0 (0.0%) | 0 (0.0%) | >0.99 |
|  | ≥IIIa | 0 (0.0%) | 0 (0.0%) | >0.99 | 0 (0.0%) | 0 (0.0%) | >0.99 |
|  | **Others complication^[[2]](#footnote-2)^** | 0 (0.0%) | 3 (0.6%) | >0.99 | 0 (0.0%) | 2 (0.9%) | >0.99 |
|  | I, II | 0 (0.0%) | 3 (0.6%) | >0.99 | 0 (0.0%) | 2 (0.9%) | >0.99 |
|  | ≥IIIa | 0 (0.0%) | 0 (0.0%) | >0.99 | 0 (0.0%) | 0 (0.0%) | >0.99 |
| 27.5 ≤ BMI < 30 |  | Before matching | | | After matching | | |
|  |  | SIDG  (N=58) | MLDG  (N=275) | *P* value | SIDG  (N=58) | MLDG  (N=95) | *P* value |
|  | The number of patients with early postoperative complication | 11 (19.0%) | 34 (12.4%) | 0.26 | 11 (19.0%) | 13 (13.7%) | 0.52 |
|  | Comprehensive Complication Index | 3.4 ± 7.8 | 2.1 ± 6.5 | 0.19 | 3.4 ± 7.8 | 2.4 ± 6.8 | 0.43 |
|  | Highest Clavien-Dindo Classification |  |  |  |  |  |  |
|  | I | 4 (6.9%) | 17 (6.2%) | >0.99 | 4 (6.9%) | 5 (5.3%) | 0.95 |
|  | II | 4 (6.9%) | 9 (3.3%) | 0.36 | 4 (6.9%) | 4 (4.2%) | 0.73 |
|  | IIIa | 3 (5.2%) | 7 (2.5%) | 0.52 | 3 (5.2%) | 4 (4.2%) | >0.99 |
|  | IIIb | 0 (0.0%) | 0 (0.0%) | >0.99 | 0 (0.0%) | 0 (0.0%) | >0.99 |
|  | IVa | 0 (0.0%) | 1 (0.4%) | >0.99 | 0 (0.0%) | 0 (0.0%) | >0.99 |
|  | IVb | 0 (0.0%) | 0 (0.0%) | >0.99 | 0 (0.0%) | 0 (0.0%) | >0.99 |
|  | V | 0 (0.0%) | 0 (0.0%) | >0.99 | 0 (0.0%) | 0 (0.0%) | >0.99 |
|  | Highest Clavien-Dindo Classification |  |  |  |  |  |  |
|  | I, II | 8 (13.8%) | 26 (9.5%) | 0.45 | 8 (13.8%) | 9 (9.5%) | 0.58 |
|  | ≥IIIa | 3 (5.2%) | 8 (2.9%) | 0.64 | 3 (5.2%) | 4 (4.2%) | >0.99 |
|  |  |  |  |  |  |  |  |
|  | **Local complication** | 5 (8.6%) | 19 (6.9%) | 0.59 | 5 (8.6%) | 6 (6.3%) | 0.75 |
|  | Wound | 0 (0.0%) | 2 (0.7%) | >0.99 | 0 (0.0%) | 1 (1.1%) | >0.99 |
|  | I, II | 0 (0.0%) | 2 (0.7%) | >0.99 | 0 (0.0%) | 1 (1.1%) | >0.99 |
|  | ≥IIIa | 0 (0.0%) | 0 (0.0%) | >0.99 | 0 (0.0%) | 0 (0.0%) | >0.99 |
|  | Fluid collection | 1 (1.7%) | 2 (0.7%) | 0.44 | 1 (1.7%) | 1 (1.1%) | >0.99 |
|  | I, II | 0 (0.0%) | 0 (0.0%) | >0.99 | 0 (0.0%) | 0 (0.0%) | >0.99 |
|  | ≥IIIa | 1 (1.7%) | 2 (0.7%) | 0.44 | 1 (1.7%) | 1 (1.1%) | >0.99 |
|  | Intra-abdominal bleeding | 1 (1.7%) | 0 (0.0%) | 0.17 | 1 (1.7%) | 0 (0.0%) | 0.38 |
|  | I, II | 0 (0.0%) | 0 (0.0%) | >0.99 | 0 (0.0%) | 0 (0.0%) | >0.99 |
|  | ≥IIIa | 1 (1.7%) | 0 (0.0%) | 0.17 | 1 (1.7%) | 0 (0.0%) | 0.38 |
|  | Intra-luminal bleeding | 0 (0.0%) | 1 (0.4%) | >0.99 | 0 (0.0%) | 0 (0.0%) | >0.99 |
|  | I, II | 0 (0.0%) | 0 (0.0%) | >0.99 | 0 (0.0%) | 0 (0.0%) | >0.99 |
|  | ≥IIIa | 0 (0.0%) | 1 (0.4%) | >0.99 | 0 (0.0%) | 0 (0.0%) | >0.99 |
|  | Motility disorder | 2 (3.4%) | 5 (1.8%) | 0.35 | 2 (3.4%) | 1 (1.1%) | 0.56 |
|  | I, II | 2 (3.4%) | 5 (1.8%) | 0.35 | 2 (3.4%) | 1 (1.1%) | 0.56 |
|  | ≥IIIa | 0 (0.0%) | 0 (0.0%) | >0.99 | 0 (0.0%) | 0 (0.0%) | >0.99 |
|  | Anastomosis stricture | 0 (0.0%) | 3 (1.1%) | >0.99 | 0 (0.0%) | 1 (1.1%) | >0.99 |
|  | I, II | 0 (0.0%) | 2 (0.7%) | >0.99 | 0 (0.0%) | 0 (0.0%) | >0.99 |
|  | ≥IIIa | 0 (0.0%) | 1 (0.4%) | >0.99 | 0 (0.0%) | 1 (1.1%) | >0.99 |
|  | Anastomosis leakage | 0 (0.0%) | 3 (1.1%) | >0.99 | 0 (0.0%) | 1 (1.1%) | >0.99 |
|  | I, II | 0 (0.0%) | 1 (0.4%) | >0.99 | 0 (0.0%) | 0 (0.0%) | >0.99 |
|  | ≥IIIa | 0 (0.0%) | 2 (0.7%) | >0.99 | 0 (0.0%) | 1 (1.1%) | >0.99 |
|  | Stump leakage | 1 (1.7%) | 1 (0.4%) | 0.32 | 1 (1.7%) | 0 (0.0%) | 0.38 |
|  | I, II | 0 (0.0%) | 0 (0.0%) | >0.99 | 0 (0.0%) | 0 (0.0%) | >0.99 |
|  | ≥IIIa | 1 (1.7%) | 1 (0.4%) | 0.32 | 1 (1.7%) | 0 (0.0%) | 0.38 |
|  | Pancreatitis | 0 (0.0%) | 2 (0.7%) | >0.99 | 0 (0.0%) | 1 (1.1%) | >0.99 |
|  | I, II | 0 (0.0%) | 2 (0.7%) | >0.99 | 0 (0.0%) | 1 (1.1%) | >0.99 |
|  | ≥IIIa | 0 (0.0%) | 0 (0.0%) | >0.99 | 0 (0.0%) | 0 (0.0%) | >0.99 |
|  | **Systemic complication** | 5 (8.6%) | 19 (6.9%) | 0.59 | 5 (8.6%) | 7 (7.4%) | 0.77 |
|  | Pulmonary | 5 (8.6%) | 15 (5.5%) | 0.36 | 5 (8.6%) | 6 (6.3%) | 0.75 |
|  | I, II | 5 (8.6%) | 14 (5.1%) | 0.35 | 5 (8.6%) | 5 (5.3%) | 0.51 |
|  | ≥IIIa | 0 (0.0%) | 1 (0.4%) | >0.99 | 0 (0.0%) | 1 (1.1%) | >0.99 |
|  | Urinary | 0 (0.0%) | 1 (0.4%) | >0.99 | 0 (0.0%) | 1 (1.1%) | >0.99 |
|  | I, II | 0 (0.0%) | 1 (0.4%) | >0.99 | 0 (0.0%) | 1 (1.1%) | >0.99 |
|  | ≥IIIa | 0 (0.0%) | 0 (0.0%) | >0.99 | 0 (0.0%) | 0 (0.0%) | >0.99 |
|  | Renal | 0 (0.0%) | 0 (0.0%) | >0.99 | 0 (0.0%) | 0 (0.0%) | >0.99 |
|  | I, II | 0 (0.0%) | 0 (0.0%) | >0.99 | 0 (0.0%) | 0 (0.0%) | >0.99 |
|  | ≥IIIa | 0 (0.0%) | 0 (0.0%) | >0.99 | 0 (0.0%) | 0 (0.0%) | >0.99 |
|  | Gastrointestinal | 0 (0.0%) | 0 (0.0%) | >0.99 | 0 (0.0%) | 0 (0.0%) | >0.99 |
|  | I, II | 0 (0.0%) | 0 (0.0%) | >0.99 | 0 (0.0%) | 0 (0.0%) | >0.99 |
|  | ≥IIIa | 0 (0.0%) | 0 (0.0%) | >0.99 | 0 (0.0%) | 0 (0.0%) | >0.99 |
|  | Hepatobiliary | 0 (0.0%) | 1 (0.4%) | >0.99 | 0 (0.0%) | 0 (0.0%) | >0.99 |
|  | I, II | 0 (0.0%) | 1 (0.4%) | >0.99 | 0 (0.0%) | 0 (0.0%) | >0.99 |
|  | ≥IIIa | 0 (0.0%) | 0 (0.0%) | >0.99 | 0 (0.0%) | 0 (0.0%) | >0.99 |
|  | Neuropsychiatric | 0 (0.0%) | 1 (0.4%) | >0.99 | 0 (0.0%) | 0 (0.0%) | >0.99 |
|  | I, II | 0 (0.0%) | 1 (0.4%) | >0.99 | 0 (0.0%) | 0 (0.0%) | >0.99 |
|  | ≥IIIa | 0 (0.0%) | 0 (0.0%) | >0.99 | 0 (0.0%) | 0 (0.0%) | >0.99 |
|  | Cardiac | 0 (0.0%) | 0 (0.0%) | >0.99 | 0 (0.0%) | 0 (0.0%) | >0.99 |
|  | I, II | 0 (0.0%) | 0 (0.0%) | >0.99 | 0 (0.0%) | 0 (0.0%) | >0.99 |
|  | ≥IIIa | 0 (0.0%) | 0 (0.0%) | >0.99 | 0 (0.0%) | 0 (0.0%) | >0.99 |
|  | Vascular | 0 (0.0%) | 2 (0.7%) | >0.99 | 0 (0.0%) | 0 (0.0%) | >0.99 |
|  | I, II | 0 (0.0%) | 2 (0.7%) | >0.99 | 0 (0.0%) | 0 (0.0%) | >0.99 |
|  | ≥IIIa | 0 (0.0%) | 0 (0.0%) | >0.99 | 0 (0.0%) | 0 (0.0%) | >0.99 |
|  | Endocrine | 0 (0.0%) | 0 (0.0%) | >0.99 | 0 (0.0%) | 0 (0.0%) | >0.99 |
|  | I, II | 0 (0.0%) | 0 (0.0%) | >0.99 | 0 (0.0%) | 0 (0.0%) | >0.99 |
|  | ≥IIIa | 0 (0.0%) | 0 (0.0%) | >0.99 | 0 (0.0%) | 0 (0.0%) | >0.99 |
|  | **Others complication^[[3]](#footnote-3)^** | 1 (1.7%) | 0 (0.0%) | 0.17 | 1 (1.7%) | 0 (0.0%) | 0.38 |
|  | I, II | 1 (1.7%) | 0 (0.0%) | 0.17 | 1 (1.7%) | 0 (0.0%) | 0.38 |
|  | ≥IIIa | 0 (0.0%) | 0 (0.0%) | >0.99 | 0 (0.0%) | 0 (0.0%) | >0.99 |
| BMI ≥ 30 |  | Before matching | | | After matching | | |
|  |  | SIDG  (N=9) | MLDG  (N=120) | *P* value | SIDG  (N=9) | MLDG  (N=28) | *P* value |
|  | The number of patients with early postoperative complication | 2 (22.2%) | 19 (15.8%) | 0.64 | 2 (22.2%) | 5 (17.9%) | >0.99 |
|  | Comprehensive Complication Index | 5.2 ± 10.5 | 3.2 ± 8.0 | 0.46 | 5.2 ± 10.5 | 3.0 ± 7.3 | 0.49 |
|  | Highest Clavien-Dindo Classification |  |  |  |  |  |  |
|  | I | 0 (0.0%) | 5 (4.2%) | >0.99 | 0 (0.0%) | 2 (7.1%) | >0.99 |
|  | II | 1 (11.1%) | 10 (8.3%) | 0.56 | 1 (11.1%) | 2 (7.1%) | >0.99 |
|  | IIIa | 1 (11.1%) | 4 (3.3%) | 0.31 | 1 (11.1%) | 1 (3.6%) | 0.43 |
|  | IIIb | 0 (0.0%) | 0 (0.0%) | >0.99 | 0 (0.0%) | 0 (0.0%) | >0.99 |
|  | IVa | 0 (0.0%) | 0 (0.0%) | >0.99 | 0 (0.0%) | 0 (0.0%) | >0.99 |
|  | IVb | 0 (0.0%) | 0 (0.0%) | >0.99 | 0 (0.0%) | 0 (0.0%) | >0.99 |
|  | V | 0 (0.0%) | 0 (0.0%) | >0.99 | 0 (0.0%) | 0 (0.0%) | >0.99 |
|  | Highest Clavien-Dindo Classification |  |  |  |  |  |  |
|  | I, II | 1 (11.1%) | 15 (12.5%) | >0.99 | 1 (11.1%) | 4 (14.3%) | >0.99 |
|  | ≥IIIa | 1 (11.1%) | 4 (3.3%) | 0.31 | 1 (11.1%) | 1 (3.6%) | 0.43 |
|  |  |  |  |  |  |  |  |
|  | **Local complication** | 0 (0.0%) | 9 (7.5%) | >0.99 | 0 (0.0%) | 4 (14.3%) | 0.55 |
|  | Wound | 0 (0.0%) | 0 (0.0%) | >0.99 | 0 (0.0%) | 0 (0.0%) | >0.99 |
|  | I, II | 0 (0.0%) | 0 (0.0%) | >0.99 | 0 (0.0%) | 0 (0.0%) | >0.99 |
|  | ≥IIIa | 0 (0.0%) | 0 (0.0%) | >0.99 | 0 (0.0%) | 0 (0.0%) | >0.99 |
|  | Fluid collection | 1 (11.1%) | 2 (1.7%) | 0.20 | 1 (11.1%) | 1 (3.6%) | 0.43 |
|  | I, II | 0 (0.0%) | 2 (1.7%) | >0.99 | 0 (0.0%) | 1 (3.6%) | >0.99 |
|  | ≥IIIa | 1 (11.1%) | 0 (0.0%) | 0.07 | 1 (11.1%) | 0 (0.0%) | 0.24 |
|  | Intra-abdominal bleeding | 0 (0.0%) | 2 (1.7%) | >0.99 | 0 (0.0%) | 1 (3.6%) | >0.99 |
|  | I, II | 0 (0.0%) | 2 (1.7%) | >0.99 | 0 (0.0%) | 1 (3.6%) | >0.99 |
|  | ≥IIIa | 0 (0.0%) | 0 (0.0%) | >0.99 | 0 (0.0%) | 0 (0.0%) | >0.99 |
|  | Intra-luminal bleeding | 0 (0.0%) | 1 (0.8%) | >0.99 | 0 (0.0%) | 0 (0.0%) | >0.99 |
|  | I, II | 0 (0.0%) | 0 (0.0%) | >0.99 | 0 (0.0%) | 0 (0.0%) | >0.99 |
|  | ≥IIIa | 0 (0.0%) | 1 (0.8%) | >0.99 | 0 (0.0%) | 0 (0.0%) | >0.99 |
|  | Motility disorder | 0 (0.0%) | 2 (1.7%) | >0.99 | 0 (0.0%) | 1 (3.6%) | >0.99 |
|  | I, II | 0 (0.0%) | 2 (1.7%) | >0.99 | 0 (0.0%) | 1 (3.6%) | >0.99 |
|  | ≥IIIa | 0 (0.0%) | 0 (0.0%) | >0.99 | 0 (0.0%) | 0 (0.0%) | >0.99 |
|  | Anastomosis stricture | 0 (0.0%) | 0 (0.0%) | >0.99 | 0 (0.0%) | 0 (0.0%) | >0.99 |
|  | I, II | 0 (0.0%) | 0 (0.0%) | >0.99 | 0 (0.0%) | 0 (0.0%) | >0.99 |
|  | ≥IIIa | 0 (0.0%) | 0 (0.0%) | >0.99 | 0 (0.0%) | 0 (0.0%) | >0.99 |
|  | Anastomosis leakage | 0 (0.0%) | 2 (1.7%) | >0.99 | 0 (0.0%) | 1 (3.6%) | >0.99 |
|  | I, II | 0 (0.0%) | 0 (0.0%) | >0.99 | 0 (0.0%) | 0 (0.0%) | >0.99 |
|  | ≥IIIa | 0 (0.0%) | 2 (1.7%) | >0.99 | 0 (0.0%) | 1 (3.6%) | >0.99 |
|  | Stump leakage | 0 (0.0%) | 0 (0.0%) | >0.99 | 0 (0.0%) | 0 (0.0%) | >0.99 |
|  | I, II | 0 (0.0%) | 0 (0.0%) | >0.99 | 0 (0.0%) | 0 (0.0%) | >0.99 |
|  | ≥IIIa | 0 (0.0%) | 0 (0.0%) | >0.99 | 0 (0.0%) | 0 (0.0%) | >0.99 |
|  | Pancreatitis | 0 (0.0%) | 0 (0.0%) | >0.99 | 0 (0.0%) | 0 (0.0%) | >0.99 |
|  | I, II | 0 (0.0%) | 0 (0.0%) | >0.99 | 0 (0.0%) | 0 (0.0%) | >0.99 |
|  | ≥IIIa | 0 (0.0%) | 0 (0.0%) | >0.99 | 0 (0.0%) | 0 (0.0%) | >0.99 |
|  | **Systemic complication** | 1 (11.1%) | 9 (7.5%) | 0.53 | 1 (11.1%) | 1 (3.6%) | 0.43 |
|  | Pulmonary | 1 (11.1%) | 5 (4.2%) | 0.36 | 1 (11.1%) | 1 (3.6%) | 0.43 |
|  | I, II | 1 (11.1%) | 5 (4.2%) | 0.36 | 1 (11.1%) | 1 (3.6%) | 0.43 |
|  | ≥IIIa | 0 (0.0%) | 0 (0.0%) | >0.99 | 0 (0.0%) | 0 (0.0%) | >0.99 |
|  | Urinary | 0 (0.0%) | 0 (0.0%) | >0.99 | 0 (0.0%) | 0 (0.0%) | >0.99 |
|  | I, II | 0 (0.0%) | 0 (0.0%) | >0.99 | 0 (0.0%) | 0 (0.0%) | >0.99 |
|  | ≥IIIa | 0 (0.0%) | 0 (0.0%) | >0.99 | 0 (0.0%) | 0 (0.0%) | >0.99 |
|  | Renal | 0 (0.0%) | 0 (0.0%) | >0.99 | 0 (0.0%) | 0 (0.0%) | >0.99 |
|  | I, II | 0 (0.0%) | 0 (0.0%) | >0.99 | 0 (0.0%) | 0 (0.0%) | >0.99 |
|  | ≥IIIa | 0 (0.0%) | 0 (0.0%) | >0.99 | 0 (0.0%) | 0 (0.0%) | >0.99 |
|  | Gastrointestinal | 0 (0.0%) | 0 (0.0%) | >0.99 | 0 (0.0%) | 0 (0.0%) | >0.99 |
|  | I, II | 0 (0.0%) | 0 (0.0%) | >0.99 | 0 (0.0%) | 0 (0.0%) | >0.99 |
|  | ≥IIIa | 0 (0.0%) | 0 (0.0%) | >0.99 | 0 (0.0%) | 0 (0.0%) | >0.99 |
|  | Hepatobiliary | 0 (0.0%) | 3 (2.5%) | >0.99 | 0 (0.0%) | 0 (0.0%) | >0.99 |
|  | I, II | 0 (0.0%) | 3 (2.5%) | >0.99 | 0 (0.0%) | 0 (0.0%) | >0.99 |
|  | ≥IIIa | 0 (0.0%) | 0 (0.0%) | >0.99 | 0 (0.0%) | 0 (0.0%) | >0.99 |
|  | Neuropsychiatric | 0 (0.0%) | 2 (1.7%) | >0.99 | 0 (0.0%) | 0 (0.0%) | >0.99 |
|  | I, II | 0 (0.0%) | 1 (0.8%) | >0.99 | 0 (0.0%) | 0 (0.0%) | >0.99 |
|  | ≥IIIa | 0 (0.0%) | 1 (0.8%) | >0.99 | 0 (0.0%) | 0 (0.0%) | >0.99 |
|  | Cardiac | 0 (0.0%) | 0 (0.0%) | >0.99 | 0 (0.0%) | 0 (0.0%) | >0.99 |
|  | I, II | 0 (0.0%) | 0 (0.0%) | >0.99 | 0 (0.0%) | 0 (0.0%) | >0.99 |
|  | ≥IIIa | 0 (0.0%) | 0 (0.0%) | >0.99 | 0 (0.0%) | 0 (0.0%) | >0.99 |
|  | Vascular | 0 (0.0%) | 0 (0.0%) | >0.99 | 0 (0.0%) | 0 (0.0%) | >0.99 |
|  | I, II | 0 (0.0%) | 0 (0.0%) | >0.99 | 0 (0.0%) | 0 (0.0%) | >0.99 |
|  | ≥IIIa | 0 (0.0%) | 0 (0.0%) | >0.99 | 0 (0.0%) | 0 (0.0%) | >0.99 |
|  | Endocrine | 0 (0.0%) | 0 (0.0%) | >0.99 | 0 (0.0%) | 0 (0.0%) | >0.99 |
|  | I, II | 0 (0.0%) | 0 (0.0%) | >0.99 | 0 (0.0%) | 0 (0.0%) | >0.99 |
|  | ≥IIIa | 0 (0.0%) | 0 (0.0%) | >0.99 | 0 (0.0%) | 0 (0.0%) | >0.99 |
|  | **Others complication^[[4]](#footnote-4)^** | 0 (0.0%) | 1 (0.8%) | >0.99 | 0 (0.0%) | 0 (0.0%) | >0.99 |
|  | I, II | 0 (0.0%) | 1 (0.8%) | >0.99 | 0 (0.0%) | 0 (0.0%) | >0.99 |
|  | ≥IIIa | 0 (0.0%) | 0 (0.0%) | >0.99 | 0 (0.0%) | 0 (0.0%) | >0.99 |

Continuous variables are presented as the mean ± standard deviation.
In the case of complication, it is expressed as the number of patients (%).

Supplementary Table 4. Clinicopathologic characteristics by sex in single-incision distal gastrectomy (SIDG) and multiport laparoscopic distal gastrectomy (MLDG) after 1:2 propensity score matching

|  | Male | | | Female | | |
| --- | --- | --- | --- | --- | --- | --- |
|  | SIDG  (N=93) | MLDG  (N=194) | *P* value | SIDG  (N=86) | MLDG  (N=164) | *P* value |
| Age (years) | 60.7 ± 10.8 | 59.6 ± 11.1 | 0.41 | 63.1 ± 11.9 | 63.9 ± 11.4 | 0.58 |
| Height (cm) | 167.1 ± 5.9 | 167.4 ± 5.8 | 0.71 | 154.0 ± 5.9 | 153.3 ± 6.6 | 0.39 |
| Weight (kg) | 75.5 ± 7.6 | 75.2 ± 7.0 | 0.74 | 65.0 ± 6.2 | 64.4 ± 7.8 | 0.51 |
| BMI (kg/m^2^) | 27.0 ± 1.8 | 26.8 ± 1.6 | 0.35 | 27.4 ± 1.7 | 27.3 ± 2.1 | 0.94 |
| ASA classification |  |  | 0.36 |  |  | >0.99 |
| I | 23 (24.7%) | 59 (30.4%) |  | 31 (36.0%) | 47 (28.7%) |  |
| II | 61 (65.6%) | 122 (62.9%) |  | 50 (58.1%) | 108 (65.9%) |  |
| III | 9 (9.7%) | 11 (5.7%) |  | 5 (5.8%) | 9 (5.5%) |  |
| IV | 0 (0.0%) | 2 (1.0%) |  | 0 (0.0%) | 0 (0.0%) |  |
| Year of operation |  |  | 0.85 |  |  | 0.73 |
| 2011 | 0 (0.0%) | 0 (0.0%) |  | 0 (0.0%) | 0 (0.0%) |  |
| 2012 | 2 (2.2%) | 1 (0.5%) |  | 1 (1.2%) | 4 (2.4%) |  |
| 2013 | 4 (4.3%) | 12 (6.2%) |  | 4 (4.7%) | 10 (6.1%) |  |
| 2014 | 3 (3.2%) | 8 (4.1%) |  | 5 (5.8%) | 6 (3.7%) |  |
| 2015 | 9 (9.7%) | 31 (16.0%) |  | 15 (17.4%) | 26 (15.9%) |  |
| 2016 | 13 (14.0%) | 25 (12.9%) |  | 17 (19.8%) | 33 (20.1%) |  |
| 2017 | 13 (14.0%) | 27 (13.9%) |  | 16 (18.6%) | 22 (13.4%) |  |
| 2018 | 12 (12.9%) | 22 (11.3%) |  | 8 (9.3%) | 23 (14.0%) |  |
| 2019 | 15 (16.1%) | 24 (12.4%) |  | 6 (7.0%) | 18 (11.0%) |  |
| 2020 | 17 (18.3%) | 35 (18.0%) |  | 9 (10.5%) | 18 (11.0%) |  |
| 2021 | 5 (5.4%) | 9 (4.6%) |  | 5 (5.8%) | 4 (2.4%) |  |
| Tumor size (cm) | 2.9 ± 1.8 | 2.6 ± 1.7 | 0.24 | 2.4 ± 1.1 | 2.8 ± 1.7 | 0.05 |
| Proximal resection margin (cm) | 5.0 ± 2.8 | 4.6 ± 2.7 | 0.16 | 4.3 ± 2.7 | 4.5 ± 2.8 | 0.65 |
| Distal resection margin (cm) | 4.8 ± 3.2 | 5.1 ± 2.7 | 0.45 | 5.6 ± 3.1 | 5.4 ± 3.0 | 0.65 |
| Number of retrieved lymph nodes | 53.4 ± 20.0 | 54.1 ± 20.5 | 0.77 | 52.1 ± 18.7 | 53.6 ± 21.8 | 0.60 |
| Number of positive lymph nodes | 0.4 ± 1.1 | 0.4 ± 1.0 | 0.99 | 0.4 ± 1.3 | 0.4 ± 1.5 | 0.98 |
| Lymphatic invasion | 18 (19.4%) | 30 (15.5%) | 0.51 | 13 (15.1%) | 30 (18.3%) | 0.65 |
| Venous invasion | 0 (0.0%) | 2 (1.0%) | 0.82 | 1 (1.2%) | 4 (2.4%) | 0.83 |
| Perineural invasion | 5 (5.4%) | 8 (4.1%) | 0.87 | 4 (4.7%) | 4 (2.4%) | 0.57 |
| Pathologic T stage |  |  | 0.84 |  |  | 0.79 |
| pT1a | 47 (50.5%) | 102 (52.6%) |  | 47 (54.7%) | 94 (57.3%) |  |
| pT1b | 46 (49.5%) | 92 (47.4%) |  | 39 (45.3%) | 70 (42.7%) |  |
| Pathologic N stage |  |  | 0.91 |  |  | 0.85 |
| pN0 | 76 (81.7%) | 156 (80.4%) |  | 72 (83.7%) | 142 (86.6%) |  |
| pN1 | 12 (12.9%) | 27 (13.9%) |  | 10 (11.6%) | 14 (8.5%) |  |
| pN2 | 5 (5.4%) | 10 (5.2%) |  | 2 (2.3%) | 5 (3.0%) |  |
| pN3a | 0 (0.0%) | 1 (0.5%) |  | 2 (2.3%) | 3 (1.8%) |  |
| Pathologic TNM stage |  |  | 0.75 |  |  | 0.53 |
| Stage I | 77 (82.8%) | 165 (85.1%) |  | 74 (86.0%) | 147 (89.6%) |  |
| Stage II | 16 (17.2%) | 29 (14.9%) |  | 12 (14.0%) | 17 (10.4%) |  |

Continuous variables are presented as the mean ± standard deviation.

Supplementary Table 5. Surgical outcomes and postoperative course by sex in single-incision distal gastrectomy (SIDG) and multiport laparoscopic distal gastrectomy (MLDG) after 1:2 propensity score matching

|  | Male | | | Female | | |
| --- | --- | --- | --- | --- | --- | --- |
|  | SIDG  (N=93) | MLDG  (N=194) | *P* value | SIDG  (N=86) | MLDG  (N=164) | *P* value |
| Operation time (minutes) | 176.4 ± 63.5 | 191.4 ± 51.8 | 0.049 | 164.7 ± 55.6 | 179.8 ± 53.1 | 0.04 |
| Intraoperative transfusion | 0 (0.0%) | 1 (0.5%) | >0.99 | 0 (0.0%) | 0 (0.0%) | >0.99 |
| Concomitant prophylactic cholecystectomy and appendectomy | 5 (5.4%) | 5 (2.6%) | 0.39 | 1 (1.2%) | 5 (3.0%) | 0.62 |
| Anastomosis |  |  | 0.14 |  |  | 0.045 |
| Billroth I | 27 (29.0%) | 42 (21.6%) |  | 16 (18.6%) | 46 (28.0%) |  |
| Billroth II | 21 (22.6%) | 34 (17.5%) |  | 17 (19.8%) | 44 (26.8%) |  |
| Roux-en-Y | 45 (48.4%) | 118 (60.8%) |  | 53 (61.6%) | 74 (45.1%) |  |
| Length of postoperative hospital stay (days) | 5.7 ± 2.0 | 6.4 ± 5.0 | 0.12 | 6.1 ± 4.5 | 6.3 ± 5.3 | 0.73 |

Continuous variables are presented as the mean ± standard deviation.

Supplementary Table 6. Postoperative morbidity and mortality within 1 month by sex in single-incision distal gastrectomy (SIDG) and multiport laparoscopic distal gastrectomy (MLDG) after 1:2 propensity score matching

|  | Male | | | Female | | |
| --- | --- | --- | --- | --- | --- | --- |
|  | SIDG  (N=93) | MLDG  (N=194) | *P* value | SIDG  (N=86) | MLDG  (N=164) | *P* value |
| The number of patients with early postoperative complication | 13 (14.0%) | 29 (14.9%) | 0.97 | 11 (12.8%) | 17 (10.4%) | 0.71 |
| Comprehensive Complication Index | 2.5 ± 7.0 | 3.0 ± 7.7 | 0.63 | 2.7 ± 8.4 | 1.9 ± 6.5 | 0.47 |
| Highest Clavien-Dindo Classification |  |  | 0.93 |  |  | 0.81 |
| I | 80 (86.0%) | 165 (85.1%) |  | 75 (87.2%) | 147 (89.6%) |  |
| II | 5 (5.4%) | 8 (4.1%) |  | 4 (4.7%) | 7 (4.3%) |  |
| IIIa | 5 (5.4%) | 14 (7.2%) |  | 3 (3.5%) | 6 (3.7%) |  |
| IIIb | 3 (3.2%) | 7 (3.6%) |  | 3 (3.5%) | 2 (1.2%) |  |
| IVa | 0 (0.0%) | 0 (0.0%) |  | 0 (0.0%) | 1 (0.6%) |  |
| IVb | 0 (0.0%) | 0 (0.0%) |  | 0 (0.0%) | 0 (0.0%) |  |
| V | 0 (0.0%) | 0 (0.0%) |  | 0 (0.0%) | 0 (0.0%) |  |
| Highest Clavien-Dindo Classification |  |  | >0.99 |  |  | 0.67 |
| I, II | 10 (10.8%) | 22 (11.3%) |  | 7 (8.1%) | 13 (7.9%) |  |
| ≥IIIa | 3 (3.2%) | 7 (3.6%) |  | 4 (4.7%) | 4 (2.4%) |  |
| **Local complication** | 6 (6.5%) | 13 (6.7%) | >0.99 | 6 (7.0%) | 9 (5.5%) | 0.78 |
| Wound | 0 (0.0%) | 0 (0.0%) | >0.99 | 1 (1.2%) | 1 (0.6%) | >0.99 |
| I, II | 0 (0.0%) | 0 (0.0%) | >0.99 | 1 (1.2%) | 1 (0.6%) | >0.99 |
| ≥IIIa | 0 (0.0%) | 0 (0.0%) | >0.99 | 0 (0.0%) | 0 (0.0%) | >0.99 |
| Fluid collection | 1 (1.1%) | 3 (1.5%) | >0.99 | 1 (1.2%) | 2 (1.2%) | >0.99 |
| I, II | 0 (0.0%) | 1 (0.5%) | >0.99 | 0 (0.0%) | 2 (1.2%) | 0.55 |
| ≥IIIa | 1 (1.1%) | 2 (1.0%) | >0.99 | 1 (1.2%) | 0 (0.0%) | 0.34 |
| Intra-abdominal bleeding | 1 (1.1%) | 0 (0.0%) | 0.32 | 0 (0.0%) | 2 (1.2%) | 0.55 |
| I, II | 0 (0.0%) | 0 (0.0%) | >0.99 | 0 (0.0%) | 1 (0.6%) | >0.99 |
| ≥IIIa | 1 (1.1%) | 0 (0.0%) | 0.32 | 0 (0.0%) | 1 (0.6%) | >0.99 |
| Intra-luminal bleeding | 0 (0.0%) | 0 (0.0%) | >0.99 | 0 (0.0%) | 0 (0.0%) | >0.99 |
| I, II | 0 (0.0%) | 0 (0.0%) | >0.99 | 0 (0.0%) | 0 (0.0%) | >0.99 |
| ≥IIIa |  |  |  |  |  |  |
| Motility disorder | 4 (4.3%) | 6 (3.1%) | 0.73 | 0 (0.0%) | 2 (1.2%) | 0.55 |
| I, II | 4 (4.3%) | 6 (3.1%) | 0.73 | 0 (0.0%) | 2 (1.2%) | 0.55 |
| ≥IIIa | 0 (0.0%) | 0 (0.0%) | >0.99 | 0 (0.0%) | 0 (0.0%) | >0.99 |
| Anastomosis stricture | 1 (1.1%) | 0 (0.0%) | 0.32 | 2 (2.3%) | 1 (0.6%) | 0.27 |
| I, II | 0 (0.0%) | 0 (0.0%) | >0.99 | 1 (1.2%) | 0 (0.0%) | 0.34 |
| ≥IIIa | 1 (1.1%) | 0 (0.0%) | 0.32 | 1 (1.2%) | 1 (0.6%) | >0.99 |
| Anastomosis leakage | 0 (0.0%) | 3 (1.5%) | 0.55 | 1 (1.2%) | 1 (0.6%) | >0.99 |
| I, II | 0 (0.0%) | 1 (0.5%) | >0.99 | 0 (0.0%) | 0 (0.0%) | >0.99 |
| ≥IIIa | 0 (0.0%) | 2 (1.0%) | >0.99 | 1 (1.2%) | 1 (0.6%) | >0.99 |
| Stump leakage | 0 (0.0%) | 1 (0.5%) | >0.99 | 1 (1.2%) | 0 (0.0%) | 0.34 |
| I, II | 0 (0.0%) | 1 (0.5%) | >0.99 | 0 (0.0%) | 0 (0.0%) | >0.99 |
| ≥IIIa | 0 (0.0%) | 0 (0.0%) | >0.99 | 1 (1.2%) | 0 (0.0%) | 0.34 |
| Pancreatitis | 1 (1.1%) | 1 (0.5%) | 0.54 | 0 (0.0%) | 0 (0.0%) | >0.99 |
| I, II | 1 (1.1%) | 1 (0.5%) | 0.54 | 0 (0.0%) | 0 (0.0%) | >0.99 |
| ≥IIIa | 0 (0.0%) | 0 (0.0%) | >0.99 | 0 (0.0%) | 0 (0.0%) | >0.99 |
| **Systemic complication** | 5 (5.4%) | 16 (8.2%) | 0.47 | 6 (7.0%) | 8 (4.9%) | 0.57 |
| Pulmonary | 5 (5.4%) | 12 (6.2%) | >0.99 | 6 (7.0%) | 8 (4.9%) | 0.57 |
| I, II | 5 (5.4%) | 11 (5.7%) | >0.99 | 5 (5.8%) | 7 (4.3%) | 0.76 |
| ≥IIIa | 0 (0.0%) | 1 (0.5%) | >0.99 | 1 (1.2%) | 1 (0.6%) | >0.99 |
| Urinary | 0 (0.0%) | 2 (1.0%) | >0.99 | 0 (0.0%) | 0 (0.0%) | >0.99 |
| I, II | 0 (0.0%) | 2 (1.0%) | >0.99 | 0 (0.0%) | 0 (0.0%) | >0.99 |
| ≥IIIa | 0 (0.0%) | 0 (0.0%) | >0.99 | 0 (0.0%) | 0 (0.0%) | >0.99 |
| Renal | 0 (0.0%) | 1 (0.5%) | >0.99 | 0 (0.0%) | 0 (0.0%) | >0.99 |
| I, II | 0 (0.0%) | 0 (0.0%) | >0.99 | 0 (0.0%) | 0 (0.0%) | >0.99 |
| ≥IIIa | 0 (0.0%) | 1 (0.5%) | >0.99 | 0 (0.0%) | 0 (0.0%) | >0.99 |
| Gastrointestinal | 0 (0.0%) | 1 (0.5%) | >0.99 | 0 (0.0%) | 0 (0.0%) | >0.99 |
| I, II | 0 (0.0%) | 1 (0.5%) | >0.99 | 0 (0.0%) | 0 (0.0%) | >0.99 |
| ≥IIIa | 0 (0.0%) | 0 (0.0%) | >0.99 | 0 (0.0%) | 0 (0.0%) | >0.99 |
| Hepatobiliary | 0 (0.0%) | 1 (0.5%) | >0.99 | 0 (0.0%) | 0 (0.0%) | >0.99 |
| I, II | 0 (0.0%) | 0 (0.0%) | >0.99 | 0 (0.0%) | 0 (0.0%) | >0.99 |
| ≥IIIa | 0 (0.0%) | 1 (0.5%) | >0.99 | 0 (0.0%) | 0 (0.0%) | >0.99 |
| Neuropsychiatric | 0 (0.0%) | 0 (0.0%) | >0.99 | 0 (0.0%) | 0 (0.0%) | >0.99 |
| I, II | 0 (0.0%) | 0 (0.0%) | >0.99 | 0 (0.0%) | 0 (0.0%) | >0.99 |
| ≥IIIa | 0 (0.0%) | 0 (0.0%) | >0.99 | 0 (0.0%) | 0 (0.0%) | >0.99 |
| Cardiac | 0 (0.0%) | 0 (0.0%) | >0.99 | 0 (0.0%) | 0 (0.0%) | >0.99 |
| I, II | 0 (0.0%) | 0 (0.0%) | >0.99 | 0 (0.0%) | 0 (0.0%) | >0.99 |
| ≥IIIa | 0 (0.0%) | 0 (0.0%) | >0.99 | 0 (0.0%) | 0 (0.0%) | >0.99 |
| Vascular | 0 (0.0%) | 0 (0.0%) | >0.99 | 0 (0.0%) | 0 (0.0%) | >0.99 |
| I, II | 0 (0.0%) | 0 (0.0%) | >0.99 | 0 (0.0%) | 0 (0.0%) | >0.99 |
| ≥IIIa | 0 (0.0%) | 0 (0.0%) | >0.99 | 0 (0.0%) | 0 (0.0%) | >0.99 |
| Endocrine | 0 (0.0%) | 0 (0.0%) | >0.99 | 0 (0.0%) | 0 (0.0%) | >0.99 |
| I, II | 0 (0.0%) | 0 (0.0%) | >0.99 | 0 (0.0%) | 0 (0.0%) | >0.99 |
| ≥IIIa | 0 (0.0%) | 0 (0.0%) | >0.99 | 0 (0.0%) | 0 (0.0%) | >0.99 |
| **Others complication^[[5]](#footnote-5)^** | 1 (1.1%) | 2 (1.0%) | >0.99 | 0 (0.0%) | 0 (0.0%) | >0.99 |
| I, II | 1 (1.1%) | 2 (1.0%) | >0.99 | 0 (0.0%) | 0 (0.0%) | >0.99 |
| ≥IIIa | 0 (0.0%) | 0 (0.0%) | >0.99 | 0 (0.0%) | 0 (0.0%) | >0.99 |

Continuous variables are presented as the mean ± standard deviation.
In the case of complication, it is expressed as the number of patients (%).

1. ASA: American Society of Anesthesiologists [↑](#footnote-ref-1)
2. Other complications: Fever of unknown origin (3), grade II. [↑](#footnote-ref-2)
3. Other complications: Fever of unknown origin (1), grade II. [↑](#footnote-ref-3)
4. Other complications: Fever of unknown origin (1), grade II. [↑](#footnote-ref-4)
5. Other complications: Fever of unknown origin (3), all grade II. [↑](#footnote-ref-5)
